# Neratinib could be effective as monotherapy or in combination with trastuzumab in HER2-low breast cancer cells and organoid models

**DOI:** 10.1038/s41416-024-02665-z

**Published:** 2024-04-10

**Authors:** Maryam Arshad, Abul Azad, Phoebe Yuen Ka Chan, Vasanthy Vigneswara, Katharina Feldinger, Siti Norasikin Mohd Nafi, Eloise Laporte-Maguire, Carmela De Santo, Jianmin Zuo, Abeer M. Shaaban, Anthony Kong

**Affiliations:** 1 Comprehensive Cancer Centre, School of Cancer and Pharmaceutical Sciences, King’s College London, London, SE1 1UL UK; 2 Institute of Cancer and Genomic Sciences, University of Birmingham, Edgbaston, B15 2TT UK; 3 Previous association, Department of Molecular Oncology, The Weatherall Institute of Molecular Medicine, University of Oxford, Oxford, OX3 9DS UK; 4 Department of Pathology, School of Medical Sciences, Universiti Sains Malaysia, Health Campus, 16150 Kota Bharu, Kelantan Malaysia; 5 Institute of Immunology and Immunotherapy, University of Birmingham, Edgbaston, B15 2TT UK; 6 Department of cellular pathology, Queen Elizabeth Hospital Birmingham, Birmingham, UK

**Keywords:** Breast cancer, Targeted therapies

## Abstract

**Background:**

Previous studies have suggested that patients with HER2-low breast cancers do not benefit from trastuzumab treatment although the reasons remain unclear.

**Methods:**

We investigated the effect of trastuzumab monotherapy and its combination with different HER2 targeting treatments in a panel of breast cancer cell lines and patient-derived organoids (PDOs) using biochemical methods and cell viability assays.

**Results:**

Compared to sensitive HER2 over-expressing (IHC3 + ) breast cancer cells, increasing doses of trastuzumab could not achieve IC50 in MDA-MB-361 (IHC 2 + FISH + ) and MDA-MB-453 (IHC 2 + FISH-) cells which showed an intermediate response to trastuzumab. Trastuzumab treatment induced upregulation of HER ligand release, resulting in the activation of HER receptors in these cells, which could account for their trastuzumab insensitivity. Adding a dual ADAM10/17 inhibitor to inhibit the shedding of HER ligands in combination with trastuzumab only showed a modest decrease in the cell viability of HER2-low breast cancer cells and PDOs. However, the panHER inhibitor neratinib was an effective monotherapy in HER2-low breast cancer cells and PDOs, and showed additive effects when combined with trastuzumab.

**Conclusion:**

This study demonstrates that neratinib in combination with trastuzumab may be effective in a subset of HER2-low breast cancers although further validation is required in a larger panel of PDOs and in future clinical studies.

## Introduction

In the era of precision medicine, Human Epidermal Growth Factor Receptor 2 (HER2*)* is the most important predictive and prognostic biomarker in breast cancer [1]. The assessment of HER2 status by immunohistochemistry (IHC) and/or in situ hybridization (ISH) is clinically essential to select appropriate patients for trastuzumab and/or other anti-HER2 treatments as guided by ASCO (American Society of Clinical Oncology) [2]. Patients with HER2 positive tumours (defined as IHC 3+ and/or HER2 copy number ≥ 6 if FISH/CEP17 ratio <2 and/or HER2 copy number ≥ 4 if FISH/CEP17 ratio ≥ 2) are usually recommended for treatment with trastuzumab and other anti-HER2 therapies. It was previously shown that HER2-positive (IHC 3 + ) tumours were more likely to respond to trastuzumab and chemotherapy than those tumours with IHC 2+ or lower ( < 2) scores [3]. However, there are some discrepancies in determining HER2 status by either IHC or FISH due to the use of different antibodies, staining platforms, and variation in cut off values for positivity [4]. The discrepancies have since improved with the introduction of the recent 2018 ASCO/CAP HER2 testing guideline. Due to this ambiguity in the classification of HER2 status previously, some patients with HER2-low tumours were found to benefit from anti-HER2 treatments in earlier studies. Paik et al. (2008) demonstrated that some patients with tumours originally identified as HER2 positive but later defined as HER2 negative by IHC/FISH score, also had improved survival outcomes from adjuvant trastuzumab containing treatment [5]. Moreover, the recent DESTINY-Breast04 trial showed that trastuzumab deruxtecan resulted in significantly longer progression-free and overall survival compared to the physician’s choice of chemotherapy in patients with HER2-low metastatic breast cancer [6]. Therefore, in view of the original threshold was set for trastuzumab treatment [7, 8] but not for the other anti-HER2 treatments or their combination, it is likely that HER2-low tumours may benefit from other FDA approved anti-HER2 treatments in addition to trastuzumab deruxtecan.

Currently, the monoclonal antibodies trastuzumab (Herceptin), pertuzumab (Perjeta), and trastuzumab emtansine (T-DM1, Kadcyla) have FDA approval for treatment of HER2-positive breast cancer in the neoadjuvant, adjuvant and metastatic settings [9]. Lapatinib, a reversible tyrosine kinase inhibitor (TKI) to HER2 and Epidermal Growth Factor Receptor (EGFR), is currently approved for use in HER2-positive breast cancer patients after progression on a trastuzumab containing regimen [10]. Other FDA approved anti-HER2 treatments include trastuzumab deruxtecan (TDxd, Enhertu), margetuximab, tucatinib and neratinib [9]. Neratinib is an irreversible TKI that interacts with the catalytic domain of the EGFR family; ErbB1, ErbB2 and ErbB4 and inhibits the tyrosine kinase activity of these receptors. Neratinib has previously displayed very potent anti-tumour activity by significantly inhibiting proliferation in HER2-positive breast cancer cells and xenograft models [11, 12]. Neratinib in combination with capecitabine is approved for use in metastatic HER2-positive breast cancer since the NALA trial showed a significant improvement in progression-free survival (PFS) and time to intervention for central nervous system disease compared to lapatinib and capecitabine [13, 14]. Furthermore, the data from the ExteNET clinical trial confirmed that neratinib significantly improved invasive disease-free survival when given as a year extended adjuvant therapy after chemotherapy and trastuzumab-based adjuvant therapy in HER2-positive breast cancer [15, 16]. Despite the increase in grade 3 diarrhoea (40% without diarrhoea prophylaxis) in neratinib arm, there was no evidence of long-term adverse consequences of neratinib-associated diarrhoea with neratinib compared with placebo [15, 16]. In addition, an increased pathological response rate was seen with the combination of trastuzumab plus neratinib with chemotherapy compared to either agent with chemotherapy in locally advanced HER2-positive breast cancer patients [17]. Moreover, clinical benefits were also observed in locally advanced or metastatic trastuzumab pre-treated HER2-positive breast cancers with the combination of neratinib and trastuzumab [18]. Neratinib was also shown to be effective in HER2 mutated non-HER2 amplified breast cancer cells [19]. However, there has been no reported evidence of effect of neratinib in HER2-low and non-HER2 mutated breast cancers.

Among the 22 identified human ADAM (a disintegrin and metalloproteinase) family, ADAM 10 and 17 are the most studied members, which regulate HER receptor signalling through the shedding of HER ligands and/or receptors [20]. We previously reported that ADAM10 and ADAM17 metalloproteases mediate resistance to trastuzumab treatment via shedding of HER ligands and the activation of HER receptors in HER2-positive breast cancer cells [21, 22]. However, the roles of ADAM10 and 17 proteases were not investigated in relation to trastuzumab treatment in HER2-low breast cancer cells.

The objective of our study was to investigate whether HER receptor activation occurs in response to trastuzumab treatment mediated by ADAM10/17 ligand-release in HER2-low breast cancer cells. We will assess the possibility of improving treatment efficacy in HER2-low breast cancer cells and patient-derived organoids (PDOs) [23] by either a) targeting ADAM proteases using an ADAM10/17 inhibitor or b) inhibiting HER receptor activation and dimerization using neratinib and/or pertuzumab in combination with trastuzumab.

## Results

### Trastuzumab sensitivity is dependent on HER2 status and HER2-low breast cancer cells are not sensitive to trastuzumab

We determined the HER2 status of a panel of different breast cancer cell lines by IHC and FISH, which revealed considerable heterogeneity amongst cell lines (Fig. S1A, S1B). Both SK-BR-3 and BT474 showed strong IHC staining 3+ and increased HER2 gene amplification, whereas BT20 and MCF-7 cells showed weak to undetectable IHC staining (1^+^ and 0) and a normal FISH status. The other four cell lines (MDA-MB-361, HCC-1569, ZR-75-1, MDA-MB-453) had an intermediate IHC 2+ staining and among these cell lines MDA-MB-361 and HCC1569 had a FISH ratio of ≥ 2 and therefore were considered as HER2 positive for FISH amplification. Interestingly, MDA-MB-453 showed weaker IHC staining than ZR-75-1 but slightly higher FISH/CEP17 ratio (Fig. S1A, S1B).

The growth inhibitory effects of anti-HER2 treatment in IHC 2+ breast cancer cell lines were assessed via cell viability and compared to anti-HER2 treatments in IHC 3 + SK-BR-3 and BT474 cells (Fig. S1C). The MCF-7 cells that express HER2 at a negligible level were used as a negative control when necessary. Increased trastuzumab dosages showed no effect on cell viability in MCF7 (IHC 0) and BT20 cells (IHC 1 + ) (Fig. S1C). Trastuzumab, on the other hand, resulted in a slight to moderate reduction in cell viability in four HER2 IHC2+ breast cancer cell lines compared to IHC 3 + BT474 and SKBR3 cells that were sensitive to trastuzumab (IC50 < 10 μg/ml) (Figure S1C). Due to intermediate response to trastuzumab, MDA-MB-361 (IHC HER2 2 + /FISH HER2 + ) and MDA-MB-453 (IHC HER2 2 + /FISH HER2-) breast cancer cell lines were chosen for our subsequent studies.

### Effect of ADAM10/17 inhibitor on MDA-MB-361 (IHC HER2 2 + /FISH + ) and MDA-MB-453 (IHC HER2 2 + /FISH-)

**Fig. 1:**
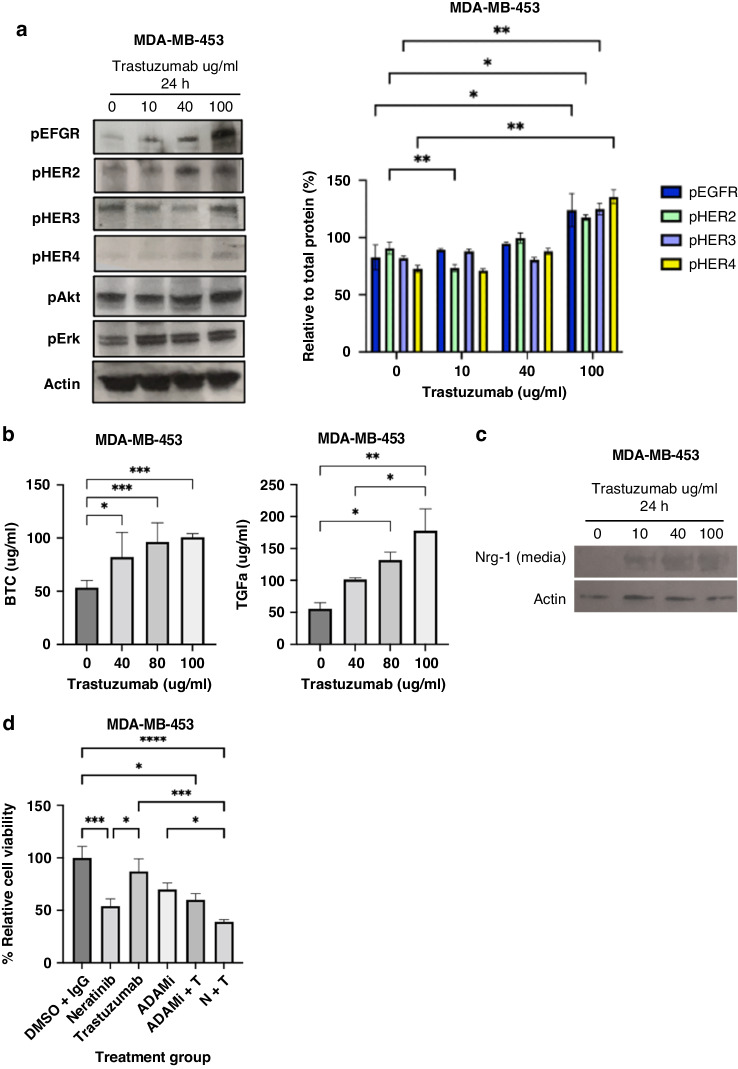
Trastuzumab induces activation of HER receptors as well as ADAMs and ligands. **A** MDA-MB-453 cells were treated with increasing doses of trastuzumab and control (IgG) for 24 h and cell lysates used for western blot and quantification of blots from three independent experiments is shown as indicated (phosphorylated proteins relative to the respective total proteins). Two-way Anova with Turkey multiple comparison test was done to determine statistically significant changes represented as *p ≤ 0.05 and **p ≤ 0.01. **B** MDA-MB-453 and MDA-MB361 cells were given same treatment as indicated, media was concentrated and used for ELISA and (**C**) western blot as indicated. **D** For cell viability experiment, 10,000 cells were seeded per well in 96 well plate and left to settle overnight before being treated with 40 µg/ml of trastuzumab, 100 nM of the anti-ADAM10/17 inhibitor, 10 nM of neratinib and or the indicated combination for 5 days in serum-reduced media. Differences of the treatment groups in comparison to control were analysed using Kruskal-Wallis-Test and *p*-values are denoted as *p ≤ 0.05, **p ≤ 0.01 and ***p ≤ 0.001.

We assessed the effect of an increasing dose of trastuzumab for 24 h in HER2-low MDA-MB-453 cells to investigate the reason of their insensitivity to trastuzumab. We found an increased activation of EGFR and HER2 as well as HER3 and HER4 receptors at higher concentrations of trastuzumab (especially 100ug/ml which was statistically significant) in MDA-MB-453 cells compared to the control group (UT) (Fig. 1A). To investigate whether the HER receptor activation is ligand-dependent, we assessed the levels of HER ligands in the media of MDA-MB-453 cells treated with an increasing dose of trastuzumab. We showed an increase in several HER ligands (NRG-1/heregulin, betacellulin and TGF-α) with an increased concentration of trastuzumab treatment for 24 h (Fig. 1B, C). Our laboratory has previously established ADAM10 and ADAM17 as the two important metalloproteases that cleave HER ligands and counteract the action of trastuzumab in HER2 overexpressing BT474 and SK-BR-3 cells [21, 22]. Therefore, we proceeded to assess the effect of a dual ADAM 10/17 inhibitor (INCB7839) in HER2-low MDA-MB-453 breast cancer cells. We showed that the dual ADAM10/17 inhibitor monotherapy slightly decreased the cell viability to 70% compared to the control and a minimally additive effect was seen when treated in combination with trastuzumab (Fig. 1D). However, the treatment effect was moderate, insufficient to decrease the cell viability below 50%, and this was not statistically significant.

Thus, our results indicate that trastuzumab induces the release of HER ligands and activation of HER receptors and trastuzumab monotherapy is not effective in decreasing cell growth in HER2-low MDA-MB-453 breast cancer cells. The dual ADAM10/17 inhibitor as a monotherapy or in combination with trastuzumab showed only a slight decrease in the cell viability of these HER2-low breast cancer cells compared to trastuzumab alone.

### PanHER inhibitor neratinib monotherapy is effective in trastuzumab insensitive MDA-MB-361 and MDA-MB-453 and shows additive inhibitory effect with trastuzumab

Since we showed that trastuzumab induces HER ligand release, resulting in the activation of HER receptors in MDA-MB-453 cells, we hypothesized that a panHER inhibitor such as neratinib could be effective in these cells due to its effectiveness against HER2 positive breast cancer cells [24]. Neratinib monotherapy was able to significantly inhibit cell viability compared to control or trastuzumab, and its combination with trastuzumab was also the most potent combination (Fig. 1D). Next, we examined the effect of neratinib on HER receptors and the downstream pathways in MDA-MB-361 and MDA-MB-453 cells, treated with increasing doses of neratinib for 24 h. We found decreased activation of EGFR, HER2 and HER3 at 10 nM concentrations but to a lesser extent with 5 nM and highest inhibition was seen at 30 nM (Fig. S2A and S2B). However, there were only minimal inhibitory effects of neratinib on the Akt and Erk activation pathways at 10 nM concentration, but greater inhibition was observed at 30 nM concentration (Fig. S2A, S2B). There was also a gradual decrease of total HER2 with increased concentrations of neratinib, which is consistent with a previously published report that neratinib induced HER2 ubiquitylation and endocytic degradation via its dissociation with HSP90 in HER2 positive breast cancer cells [25]. We further showed that HSP90 dissociation with HER2 was also induced by neratinib in HER2-low breast cancer cells (Fig. S2C), similar to those reported in HER2 positive breast cancer cells [25].

**Fig. 2:**
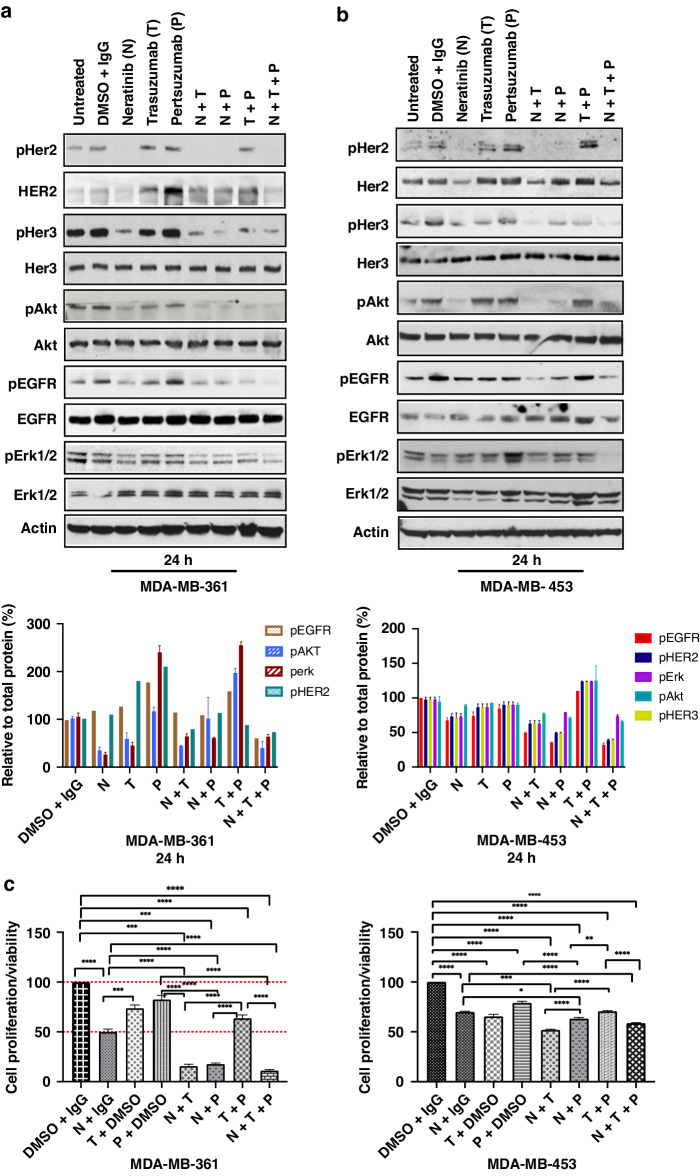
Reduced activation of HER receptors and downstream signalling proteins in MDA-MB-453 and MDA-MB361 cells with the combination anti- HER2 treatments. **A** MDA-MB-361 and (**B**) MDA-MB-453 cells were treated with 10 nM Neratinib, 40 µg/ml trastuzumab and 20 µg/ml of Pertuzumab for 24 h either alone or in combination. Cell lysates were used for western blot as indicated and quantification of four blots is shown relative to the respective total proteins. **C** MDA-MD-361 and MDA-MB-453 cells treated with 10 nM neratinib, 40 µg/ml trastuzumab and 20 µg/ml of Pertuzumab for 5 days in serum-reduced media. Cell proliferation was assessed by cell titre Glo 5 days post treatments. All Data is from 3 independent experiments with 3 technical replicates and is normalized to the control (untreated or DMSO + IgG). Graphs plotted represent mean and error bars ± SEM. Difference in the mean between groups was analysed by one-way Anova with Turkey multiple comparison test; statistically significant changes are represented by asterisks as *p ≤ 0.05, **p ≤ 0.01, ***p ≤ 0.001 and ****p < 0.0001.

Since HER ligand release would result in HER2 dimerization with other HER receptors in addition to the activation of HER receptors, we compared the effect of pertuzumab that inhibits HER2 dimerization with neratinib or trastuzumab treatment, either alone or in combination with each other. To investigate the best drug combination, we examined the effect of trastuzumab at 40 µg/ml, pertuzumab at 20 µg/ml, neratinib at 10 nM either alone or in combination with each other for 24 h on HER receptors and downstream pathways. In both cell lines, there was evidence of HER2 downregulation with neratinib or neratinib containing combinations (Figs. 2A, B). Neratinib alone or its combination with trastuzumab and/or pertuzumab decreased pHER2 in both cell lines (Fig. 2A, B). In addition, there was deactivation of EGFR, HER3 and Akt by neratinib alone or in combination with other anti-HER2 treatments, although there were differences between the two cell lines. The greatest inhibition of EGFR, HER3 and Akt activation was seen with neratinib with trastuzumab and/or pertuzumab in MDA-MB-453 cells (Fig. 2B). In MDA-MB-361 cells, neratinib alone or in any combination could decrease pEGFR, pHER3 and pAkt but the greatest inhibitory effect of pEGFR was seen with the triple combination (Fig. 2A). There was a decrease of pErk in neratinib containing treatment conditions in MDA-MB-361 cells but the greatest inhibition of Erk was observed with the triple combinations in MDA-MB-453 cells (Fig. 2A, B).

Since we were unable to observe any major differences in the inhibition of EGFR, HER2 and HER3 receptors with neratinib monotherapy at 10 nM or in combination with trastuzumab ± pertuzumab, we hypothesized that the inhibitory effect due to higher concentration of neratinib is most likely to be too effective and not enhanced by its combination with other anti-HER2 treatments. Therefore, we proceeded to investigate the effect of neratinib at a lower concentration at 5 nM alone and in combination with trastuzumab ± pertuzumab. We found that the decreased activation of EGFR, HER3 and Akt with the combination of neratinib and trastuzumab or triple combination to be more effective than neratinib 5 nM monotherapy in MDAMB-361 cells and to a lesser extent in MDA-MB-453 cells. We only observed a decrease in pErk with triple combination in both cell lines (Fig. S3A, B).

Next, we tested the effect of neratinib, pertuzumab or trastuzumab either alone or in combinations on the cell proliferation/viability. In both MDA-MB-361 and MDA-MB-453 cells, the more effective combinations were neratinib with trastuzumab or the triple combination, but neratinib with pertuzumab was also effective in FISH positive MDA-MB-361 cells (Fig. 2C, S4A, S4B, 4C). The combination of pertuzumab with trastuzumab was not effective in decreasing cell growth below 50% compared to DMSO + IgG control in both cell lines, which is in contrast with their effect on HER2 over-expressed breast cancer cell lines [26], correlated with the inability to decrease pHER2 and to downregulate HER2 at 24 h in these cells (Fig. 2A, B). Overall, neratinib and trastuzumab combination was more effective than neratinib or trastuzumab monotherapy in both cell lines, which is consistent with our biochemical data in combination with lower concentrations of neratinib at 5 nM (Fig. S3A, S3B). The additive effect of combination treatment appeared to be reduced with high doses of neratinib in MDA-MB-453 cells (Fig. S4D).

Collectively, the above observations show that neratinib but not trastuzumab, is effective in decreasing the activation of HER receptors and downstream pathways as well as inhibiting cell growth in MDA-MB-453 and MDA-MB-361 breast cancer cell lines. However, the combination of neratinib with trastuzumab (with or without pertuzumab) was more effective than neratinib monotherapy in inhibiting cell growth, consistent with greater inhibition of HER receptor activation and HER2 downregulation in both cell lines. Thus, our data provide a rational for the use of neratinib in combination with trastuzumab (with or without pertuzumab) in the treatment of HER2-low breast cancer cells.

### Comparing the effect of neratinib with tucatinib in HER2-low MDA-MB-453 cells and HER2 negative MCF-7 cells

Since tucatinib was shown to be a more HER2 selective inhibitor, we compared the effect of tucatinib with neratinib on HER receptor signalling and cell growth. At the same 10 nM concentration, we showed that neratinib was more effective than tucatinib in inhibiting pHER2, pEGFR and pHER3 at 4 h in MDA-MB-453 cells (Fig. S5A). We further compared neratinib and tucatinib at 10 nM concentration (± trastuzumab) and showed that neratinib ± trastuzumab was more effective in decreasing pHER2 than tucatinib ± trastuzumab at 24 h but the difference in inhibition was not significant for pEGFR, pHER3 and pHER4 in MDA-MB-453 cells (Fig. S5B). This is also consistent with the cell viability result with neratinib ± trastuzumab being more effective than tucatinib ± trastuzumab although the combination of neratinib with trastuzumab was the most effective among all the treatment conditions in inhibiting cell viability below 50% compared to the control (Fig. S5C). We could not detect any obvious differences between neratinib and tucatinib in inhibiting HER receptor signalling in MCF-7 cells which were only minimally responsive to either drugs or their combination with trastuzumab (Fig. S5B, Fig. S5C).

### Development of a panel of patient-derived breast cancer organoids and histological characterization

In view of the limitations of 2D cell lines models in translating laboratory findings to clinical benefit for patients, we established a panel of breast cancer patient-derived organoids (PDOs) from surgically resected breast tumours in view of the potential of PDOs in predicting treatment response in patients [23]. Using a published protocol [23], we developed a panel of breast cancer PDOs with varying HER2 and ER/PR expression (Table 1) in order to test the effects of different anti-HER2 treatments and/or a ADAM10/17 inhibitor.

**Table 1:** The histological features of the human samples from which the organoids were derived from.

Samples	Description	Histology invasive type	Tumour grade	Receptor status			Neratinib IC50 (nM)
				ER status (Quick score)	PR status (Quick score)	HER2 (score)	
T-S358174	HER2-low (previously TNBC)	Ductal (NST)	3	Negative (0/8)	Negative (0/8)	Negative (1 + )	261.8
T-S386416	HER2-low (previously HER-ve)	Ductal NST	2	Positive (7/8)	Positive (8/8)	2+ Equivocal (FISH: Neg)	154.4
T-S396391	HER2-ve	Lobular carcinoma	2	Positive (8/8)	Positive (8/8)	Negative (0)	299
T-S403276	HER2+ve	Ductal NST	2	Positive (7/8)	Positive (8/8)	Positive (2 + ) (FISH: Positive)	78.98
T-S396358	HER2-ve	Ductal NST	2	Positive (7/8)	Positive (8/8)	Negative (0)	277.4
T-S410880	HER2-ve	Ductal NST	2	Positive (4/8) (5-10%)	Positive (7/8)	Negative (0)	267.1
T-S410460	TNBC	Ductal NST	2	Negative (0/8)	Negative (0/8)	Negative (0)	226.2
T-S446358	HER2 -ve	Ductal NST	2	Positive (8/8)	Positive (8/8)	Negative (0)	Not done

The IC50 neratinib doses for the organoids are listed in the furthest right column.

*NST* invasive no special type carcinoma.

To test whether the breast cancer PDOs morphologically reflect the derived tumour, we performed histopathological analysis of H&E and IHC receptor status on the stained tissue and its corresponding organoid sections of PDOs. The phenotype of a PDO (T-S403276) showed similar morphology to the architecture of the derived tumour (Figure S6A). In addition, this breast cancer organoid represented clear cancerous features like the formation of tubules, enlarged pleomorphic nuclei and high mitotic activity as represented in H&E-stained sections (Fig. S6B). Compared with the cancerous tissue, the corresponding normal organoid (N-S403275) established from surrounding normal tissue of the tumour, displayed a well-organized structure and mildly complex cribriform architecture, and was histologically judged to display normal breast epithelium (Fig. S6A, S6C).

Beside histological conservation, PDOs tend to recapitulate the expression of some of the most important diagnostic and prognostic markers including oestrogen receptor (ER), progesterone receptor (PR), and HER2. We found that ER, PR and HER2 status was also retained in the breast cancer organoid as determined by IHC in tumour and its corresponding organoid (T-S403276). Tumour ER/PR and HER2 status corresponded to its derived organoid, and the majority of the PDO structures retained 70% of the staining (Fig. S6B). While the healthy organoids derived from the normal surrounding tissue with negative ER, PR and HER2 status also corresponded to their normal tissue (Fig. S6C). The bright field histological images of compact, coherent organoid structure of T-S403276 and the corresponding normal PDO (N-S403275) as well as another example of discohesive PDO (T-S396358) are shown in Fig. S6D.

In summary we found that the breast cancer organoid, TS403276, recapitulated its originating tumour tissue architecture and histological features, as well as hormone receptor and HER2 status.

### Effects of neratinib, trastuzumab, ADAM10/17 inhibitor in a panel of PDOs

**Fig. 3:**
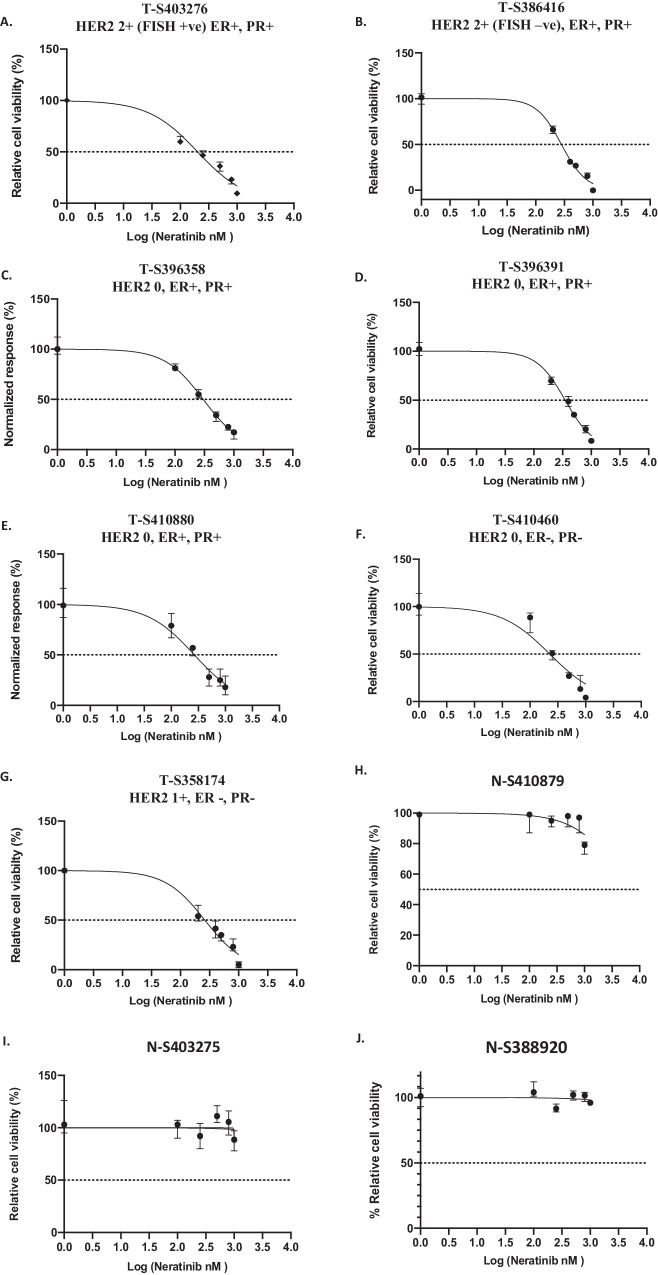
Patient-derived breast cancer organoids with different HER2 status are sensitive to neratinib. A panel of breast cancer organoids (**A**) T-S403276 (HER2 +ve, IHC 2+ and FISH +ve), (**B**) T-S386416 (HER2 equivocal, IHC 2+ and FISH -ve), (**C**) T-S396358 (HER2 -ve), (**D**) T-S396391 (HER2 -ve), (**E**) T-S410880 (HER2 –ve), (**F**) T-S410460 (TNBC) (**G**) T-S358174 (HER2-low)] and their corresponding normal organoids (**H**) N-S410879, (**I**) N-S403275 and (**J**) N-S388920) were treated with an increasing concentration of neratinib for 5 days to obtain dose response curves. Cell proliferation was assessed using 3D cell titre Glo and graph is normalized to control (DMSO + IgG). GraphPad Prism 8 software was used to plot the curves and obtain R-squared and EC50 values. Data presented from 3 independent experiments (*n* = 3) with 3 technical replicates; error bars are shown as the median ± range.

We established above that neratinib alone or in combination with trastuzumab significantly reduced the cell viability of MDA-MB-453 and MDA-MB-361 cells. Since PDOs have been shown to better recapitulate the tumour characteristics and may help to predict treatment response [23], we assessed the effects of neratinib with or without trastuzumab in a panel of PDOs. Firstly, the panel of PDOs with different HER2 and ER/PR expression were treated with the increasing doses of neratinib monotherapy for 5 days and DMSO was used as a control. We observed a dose dependent decrease in cell viability in a panel of PDOs (Fig. 3A–G), with the greatest sensitivity being observed in HER2 positive PDO (T- S403276) with an IC50 of 78.9 nM, followed by HER2 equivocal (PDO T-S386416) with an IC50 of 154.4 nM and then five HER2 IHC negative or 1+ PDOs (T-S396358, T-S396391, TS420880, TS410460 and TS358174) with IC50s 250-300 nM (Fig. 3A–G, Table 1). Furthermore, we also treated healthy organoids derived from the normal surrounding tissues with an increasing dose of neratinib (Fig. 3H–J). We observed no sensitivity of neratinib in these normal organoids even at higher dose of 1uM and no change was observed in cell viability when compared to the control group (Fig. 3H–J).

We assessed the effect of increasing doses of neratinib in combination with trastuzumab in another PDO T-S396358 (HER2 IHC 0 and ER/PR + , Table 1) and showed that trastuzumab enhanced the effect of neratinib at higher doses of 100 nM and 300 nM of neratinib (Fig. S7A). We also treated one less sensitive TNBC organoid (T-S358174, low HER2 and ER/PR negative) with high doses of neratinib and in combination with trastuzumab. The results showed that neratinib monotherapy 500 nM could decrease the cell viability to around 50% compared to the control, and the addition of trastuzumab did not result in any statistically significant difference (Fig. S7B). However, higher dose of 1uM neratinib, was toxic and displayed necrotic structures in less sensitive PDO (T-S358174), which was enhanced by trastuzumab treatment (Fig. S7C). We also compared the effect between neratinib and tucatinib in a HER2-low PDO (T-S485871, HER2 FISH 1+ and ER positive) and showed that neratinib was more effective than tucatinib (Fig. S5D).

**Fig. 4:**
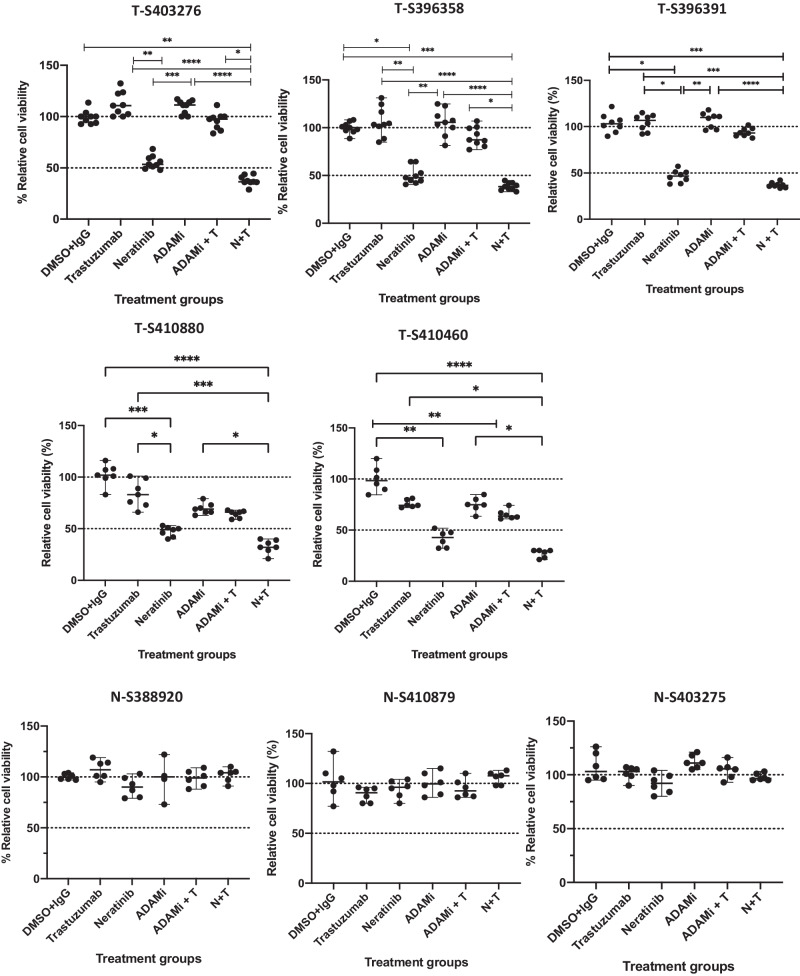
Combination of trastuzumab, neratinib, ADAM10/17 inhibitor INCB7839 or their combination in a panel of patient-derived organoids. A panel of breast cancer organoids some of their corresponding normal organoids were treated with neratinib (300 nM), trastuzumab (40 μg/ml) and ADAM10/17 inhibitor INCB7839 (300 nM) for 5 days and cell viability was assessed using 3D cell titre Glo and the results were normalized to control (DMSO + IgG). Data presented from 3 independent experiments (*n* = 3) with 3 technical replicates; data is shown as the median ± range; non-parametric Kruskal Wallis test was performed *p* values are denoted as *p ≤ 0.05, **p ≤ 0.01, ***p ≤ 0.001 and ****p < 0.0001.

In a further experiment, five additional PDOs (T-S403276, T-S396358, T-S396391, T-S420880, T-S410460) did not display any sensitivity to trastuzumab compared to the control (Fig. 4A–E). However, neratinib as a single agent at a dose of 300 nM (>IC50 doses for the five PDOs) decreased the cell viability by 50%. The addition of trastuzumab was slightly more effective than neratinib alone but this was not statistically significant compared to neratinib alone in all the PDOs (Fig. 4A–E). Furthermore, we observed no effects of any of the treatments in three normal PDOs as expected (Fig. 4F–H).

We also proceeded to investigate the effect of dual ADAM10/17 inhibition using INCB7839 in the panel of PDOs in view of the results seen in HER2-low breast cancer cells. We assessed the effects of the ADAM10/17 inhibitor together with neratinib in five PDOs, (T- S403276, T-S396358, T-S396391, T-S420880, T-S410460) (Fig. 4A–E). The treatment with the ADAM10/17 inhibitor monotherapy showed no or minimal sensitivity compared to the control, and the combination of the ADAM10/17 inhibitor and trastuzumab displayed some additive effects but were not statistically significant (Fig. 4A–E). These results are consistent with the modest inhibitory effect observed in the dual ADAM10/17 inhibitor ± trastuzumab in HER2-low breast cancer cells.

### Trastuzumab induces antibody-dependent cell cytotoxicity (ADCC) response mediated by natural killer (NK) cell in PDOs

Trastuzumab is a potent mediator of antibody-dependent cell-mediated cytotoxicity (ADCC). Studies have shown that the trastuzumab-induced ADCC response, mediated by NK cells, is preferentially exerted on HER2 overexpressing cancer cells compared to those that do not overexpress HER2 [27]. Therefore, we proceeded to investigate trastuzumab induced NK cell-mediated ADCC in two breast cancer PDOs (one was HER2 IHC 2+ and FISH positive, like in MDA-MB-361 cells; and another one IHC 0).

Firstly, the PDOs T-S403276 (HER2 IHC2+ and FISH positive, ER/PR + ) and T-S446358 (HER2 0, ER/PR + ) were successfully co-cultured with NK cells isolated from healthy donors. Furthermore, the PDOs were treated with increasing doses of trastuzumab and neratinib monotherapy for 5 days, and DMSO + IgG was used as a control. Here we observed a dose-dependent decrease in cell viability with trastuzumab or neratinib monotherapy in the presence of NK cells in both PDOs (Supplementary Fig. 8A, B). The PDO T-S403276 seemed to be more sensitive to neratinib or trastuzumab monotherapy than PDO T-S446358, showing 50% decrease in cell viability with 20 μg/ul trastuzumab and 50 nM of neratinib. In contrast, 50% decrease in cell viability was only observed in ≥100 nM neratinib and 100 μg/ul trastuzumab compared to the control group (DMSO + IgG) in T-S446358 PDO (Supplementary Fig. 8A, B).

Similarly, in a comparative study between neratinib and trastuzumab treatment groups, PDO T-S403276 displayed significant decrease in cell viability with the combination treatment, neratinib and trastuzumab, in the presence of NK cells compared to the combination treatment without NK cells (Supplementary Fig. 8C). While PDO T-S446358 still shows that the combination treatment of neratinib and trastuzumab was effective compared to trastuzuamb and neratinib monotherapy, however no significant difference was observed in the presence of NK-cells (Supplementary Fig. 8C).

In a further study, NK cells were treated with a CD16-specific blocking antibody to assess whether the anti-tumour effects of trastuzumab observed in this study were related to ADCC. We observed no significant change in cell viability in neratinib group treated with anti-CD16 compared to those without anti-CD16 (Supplementary Fig. 8D). However, in the groups of trastuzumab alone or in combination with neratinib, the anti-proliferation effect was decreased in the presence of a CD16-specific blocking antibody, suggesting a possible ADCC mechanism of response.

In summary, trastuzumab monotherapy and or neratinib monotherapy displayed a dose-dependent decrease in cell viability in the presence of NK cells. In addition, neratinib in combination with trastuzumab, was the most effective treatment in the presence of NK cells reducing the cell viability by more than 80%. Furthermore, antitumour effects of trastuzumab are mediated by NK-cell induced ADCC.

## Discussion

In the NSABP B-31 clinical trial, 174 patients with initially defined HER2 positive tumours but later found to be HER2 negative, were shown to have increased PFS and overall survival (OS) when treated with additional trastuzumab (82/174) compared to those not treated with trastuzumab (92/174) [5]. The concept of HER2-low tumours and the potential benefit from anti-HER2 therapy has recently been proposed. Therefore, there is a need to re-investigate the response of trastuzumab and other anti-HER2 agents in HER2-low breast cancer cells in order to improve in the selection of appropriate patients for these therapies. In addition, it is unclear how each of these anti-HER2 therapies may benefit different subgroup of patients. It is important to further understand the underlying response to different anti-HER2 treatments or their combination in those HER2-low breast tumours.

In our studies of trastuzumab responses in a panel of breast cancer cells, MDA-MB-453 (IHC HER2 2 + /FISH-) and MDA-MB-361 (IHC HER2 2 + /FISH + ) cells have shown only moderate responses to trastuzumab when compared to HER2 over-expressing (IHC 3 + ) BT474 and SKBR3 cells. Our laboratory has previously established ADAM10 and ADAM17 as important metalloproteinases that cleave HER ligands and counteract the action of trastuzumab in HER2 overexpressing BT474 and SKBR3 cells [22]. Therefore, the role of these two ADAMs in relation to trastuzumab treatment in MDA-MB-453 and MDA-MB-361 cells was assessed. We found that the dual ADAM10/17 inhibitor only exhibited a slight reduction in cell viability in HER2-low MDA-MB-453 cells. We also investigated the combination of trastuzumab with a dual ADAM10/17 inhibitor in these breast cancer cells and only observed minimally additive or synergistic effects, consistent with the PDOs findings.

Although several different ADAMs have been implicated in cancer initiation and progression [28], as well as conferring resistance to specific cancer therapies in various tumour types, ADAM17 and ADAM10 remain as the two most important metalloproteases [29, 30]. Upregulation of ADAM10 and 17 have been found in multiple cancers and they have been investigated as therapeutic targets [31]. One of the first inhibitors INCB3619, a dual ADAM10/17 inhibitor, was investigated and showed anti-cancer activity in animal model of breast cancer [31]. Recently, another inhibitor INCB7839 with better pharmacokinetic properties [32, 33] has undergone a clinical trial in patients with HER2-positive breast cancer. The increased clinical efficacy of INCB7839 in combination with trastuzumab was seen in subset of HER2-positive metastatic breast cancer patients [34]. Despite these initial promising findings, further clinical trials with INCB7839 in breast cancer have not been carried out. So far, all developed ADAM inhibitors failed in clinical trials because of poor efficacy or side effects [33]. Since we only tested a few breast cancer organoids, we cannot totally rule out that this inhibitor may be useful in some HER2-low breast cancer cells. Therefore, more breast organoid models need to be tested to find out which subset of tumours may gain benefit from this inhibitor with trastuzumab. Moreover, INCB7839 showed clinical responses in combination with trastuzumab in a subset of HER2-positive metastatic breast cancer patients expressing the p95 form of HER2 [34]. However, we have not tested whether the HER2-low breast cancer cells expressed p95 or not.

Since ADAM10 and 17 upregulation results in the shedding of HER ligands and dimerization of HER2 with other HER receptors and their activation [22, 32], we investigated the effect of inhibiting HER receptors with the panHER inhibitor neratinib in combination with anti-HER2 agents (trastuzumab and pertuzumab) in HER2-low breast cancer cells. Moreover, the HER2 positivity threshold was initially based on the response to trastuzumab. It is unknown whether these criteria should be different for TKIs like neratinib or in combination with different anti-HER2 treatments since many studies have suggested that HER2 negative tumours may benefit from these treatments [35]. The data from this study suggested that HER2-low breast cell lines may respond to neratinib despite showing insensitivity to trastuzumab. In our studies, we showed that compared to trastuzumab, neratinib monotherapy is slightly more effective in reducing cell viability in both cell lines by approximately 50%. However, the combination of neratinib with trastuzumab (with or without pertuzumab) showed a significant reduction in cell viability compared to either monotherapy, which is more effective in MDA-MD-361 cell lines. The reason why MDA-MA-453 is not equally responsive as MDA-MB-361could be due to the non-HER2 gene amplification as determined by FISH ratio, although both cell lines are equivocal as judged by IHC score of 2+ or may be inherently resistant to trastuzumab. This observation is also further supported by our results from the breast organoid studies in which greatest response was seen with neratinib in the HER2-positive model followed by an intermediate response with HER2 equivocal model and the lowest response was seen with HER2 negative models. Unexpectedly, we did not observe any differences in the response to trastuzumab or its combination with an ADAM inhibitor between the five organoids with different HER2 status, but further validation is required in a larger panel of breast organoids. Although we have not seen major differences between neratinib with trastuzumab and neratinib with pertuzumab combinations in the de-activation of HER2, HER3 and Akt as well as the cell viability of MDA-MD-361 cells, we have seen a greater decrease in pEGFR, pHER3, pAkt, pErk and reduction in the cell viability in MDA-MD-453 cells with neratinib and trastuzumab combination compared to neratinib with pertuzumab. Thus, neratinib and trastuzumab may be a superior combination to neratinib and pertuzumab.

The data from the NALA trial confirmed that neratinib significantly improved the PFS compared to lapatinib in metastatic breast cancer [36]. Moreover, neratinib was previously shown to induce HSP90 dissociation with HER2 leading to its ubiquitylation and endocytic degradation in HER2 positive breast cancer cells, providing an insight into a new mechanism of action in addition to the inhibition of HER receptor activation and downstream signal transduction [25]. We have also confirmed that neratinib decreased HER2 downregulation via HSP90 dissociation with HER2 in HER2-low breast cancer cells in this study. Thus, neratinib may provide a potential benefit in reducing the chance of developing acquired resistance to trastuzumab [37, 38]. Additionally, neratinib is an irreversible panHER inhibitor for all HER receptors whereas lapatinib is reversible tyrosine kinase inhibitor against EGFR and HER2. In our studies, we showed that neratinib is effective as low as 5 nM concentrations whereas lapatinib is not effective at lower concentrations in HER2 positive breast cancer cells [39]. Furthermore, we also showed greater activity of neratinib over tucatinib in HER2-low BC. Thus, neratinib might be more useful in treating HER2-positive as well HER2 equivocal breast cancers since lapatinib was found to be only sensitive for HER2-positive breast cancer cells. Recently, neratinib has been shown to act as a very potent inhibitor of different cancers with HER2 mutations compared to lapatinib [19], although resistance to neratinib could develop through the activation of mTORC pathway in these cancers [40]. We have not tested the involvement of mTORC pathway in MDA-MB-453 and MDA-MB-361cells in the current study. A subgroup analysis of the NSABP B-41 trial showed that patients with IHC 2+ tumours appeared to respond better to lapatinib and chemotherapy compared to trastuzumab and chemotherapy albeit this was not statistically significant [41], indicating a need to further assess the effect of neratinib in HER2 IHC 2+ tumours (both FISH + and FISH-) in combination with trastuzumab or other trastuzumab-based antibody conjugates in clinical trials.

In summary, we have shown that neratinib monotherapy and in combination with trastuzumab was effective in a small subset of HER2-low breast cancer cells and PDOs. Further studies are required to assess the effects of neratinib with trastuzumab in combination with chemotherapy or antibody conjugates such as TDM1 or trastuzumab deruxtecan in these HER2-low tumours. Further studies are also required to investigate other neratinib based combinations such as neratinib in combination with hormone treatments and/or CDK4/6 inhibitors in HER2-low ER-positive breast cancer cells and PDOs, as well as neratinib in combination with anti-PDL1/PD1 in HER2-low/TNBC cells and PDOs. This will help to design clinical trials to integrate neratinib in the treatment pathways of HER2-low breast cancers in the future.

## Materials and methods

### Cell culture, transfection and reagents

All cell lines were obtained from Prof. Adrian Harris’ lab at University of Oxford apart from BT474 and SKBR3 cell lines, which were previously provided by the cell services lab at Cancer Research UK (Lincoln’s Inn Fields laboratory). The relevant cell lines had been previously authenticated and cell lines were tested for mycoplasma contamination at regular intervals when they were cultured in the lab for experiments. SKBR3, BT20, MCF7, MDA-MB-453 and MDA-MB-361 cells were cultured in DMEM supplemented with 10% FCS, 1% Penicillin/Streptomycin. BT474, ZR75-1 and HCC1569 were grown in RPMI supplemented with 10% FCS, 1% Penicillin/Streptomycin. Trastuzumab and Pertuzumab were obtained from the pharmacy department in the National Health Service. Neratinib was obtained from PUMA Biotechnology (USA) and ADAM10/17 inhibitor INCB7839 was purchased from Carbosynth.

### Immunoblot and antibodies

Cell lysates were prepared for western blotting in either RIPA buffer (Pierce) or lysis buffer [20 mM Tris-HCl (pH7.5), 150 mM NaCl, 1 mM EDTA, 0.5% DOC, 0.1% SDS, 1% TritonX-100, 10% glycerol containing protease and phosphatase inhibitor cocktail (Pierce)]. Cells were centrifuged at 13,500 rpm for 10 min at 4 °C. Supernatants were collected and protein concentration was quantified using the Bradford assay. Briefly, 30–60 µg protein were separated on SDS-PAGE (Invitrogen or Bio-Rad) and transferred to nitrocellulose (Amersham) or PVDF (Bio-Rad) membrane. Standard procedures were applied for western blotting. The following antibodies were used: EGFR, pEGFR, HER4 and pHER4 were from Santa Cruz Technology; HER2, pHER2, HER3, pHER3, Akt, pAkt, ERK1/2 and pERK1/2 were from cell signalling technology; NRG was obtained from Abcam.

### Determination of HER ligands in conditioned medium by ELISA

To investigate the levels of the ligands of betacellulin (BTC) and TGFα secreted into the media, the cells were seeded in 10 cm tissue culture dishes at a density of ~1,00,000 cells in full media and were allowed to attach overnight at 37 °C and 5% CO_2_. The cells were treated with the relevant drugs for 24 h and at the end of treatment, media was collected in 15 ml tubes and centrifuged at 2300 rpm at 4 °C for 5 min. The supernatant was then transferred to Amicon Ultra centrifugal filter units (Millipore) and was centrifuged at 3200 rpm at 4 °C for 30 min. The protein concentrations were determined by BCA assay and the samples were investigated by either western blot or by Dual Set ELISA kit (R&D Systems) according to manufacturer’s instructions.

### Fluorescence in-situ hybridisation (FISH)

After optimization of cell-samples (cell pellet vs cyto-spin), pre-treatment and temperature/cycle profiles, the following procedure was used for FISH. Cyto-spin slides were fixed in acetone for 10 min. The HER2/Chromosome probe (Dako) was added, and the sample covered with a cover slip. Slides were placed in a hybridizer and left for 5 min at 82 °C (melting temperature) before the temperature decreased to 37 °C and samples were left for 48 h for hybridization. Afterwards, slides were washed in the stringent wash provided by the manufacturer first at room temperature and then at 65 °C for 10 min. After a final wash with PBS, slides were mounted using Aquatex (with DAPI). Z-stack pictures were taken and the number of green (chromosome 17) and red (HER2) dots counted and HER2: Chromosome 17copy number ratio was calculated.

### Immunohistochemistry (IHC)

For immunohistochemistry, breast cancer cell samples were first heated at 37 °C for 10 min and then placed in citroclean (twice), 100% ethanol (twice), 50% ethanol, and tap water for 5 min each. For antigen retrieval a citrate buffer, pH 6, was used. After a washing step and blocking the slides with horse serum (Vector laboratories) primary antibodies HER2, 21D3, Cell signalling at a concentration of 1.150 were applied overnight at 4 °C. The next day, slides were washed, and the appropriate secondary antibody (Vector Laboratories) was added for 30 min at room temperature, before slides were incubated with DAB, counterstained with haematoxylin solution and mounted using Aquatex.

### Histology and assessment of breast cancer organoids

Normal and tumour breast cancer organoid cells were embedded in agar followed by paraffin embedding into the cell blocks. Fresh sections were cut at 3-4 microns, stained with standard haematoxylin and eosin, and examined by a specialist breast pathologist (AMS) to assess morphological features and cellular content. IHC was performed for ER, PR (using Dako Agilent Autostainer Link for ER and PR) and HER2 (using Ventana BenchMark ULTRA autostainer) as previously described [42]. Positive and negative controls were included in each batch of IHC staining. Interpretation of staining was done following the standard pathology guidelines [43, 44].

### Flow cytometry

A quantitative assessment of drug-induced apoptosis was performed using fluorescein isothiocyanate (FITC) Annexin V Apoptosis Detection Kit (BD Pharmingen, UK). Prior to the treatments with drugs, cells were seeded in complete medium at a density of 25,000 per well in 12-well plates for allowing them to adhere and enter log phase. The cells were treated with the indicated drugs (neratinib, trastuzumab, pertuzumab or their combination) for 5 days in 2.5% serum medium. On day 3 post-treatment, the plates were spun at 1500 g for 3–5 min, allowing the dead cells to settle in the culture, old media was removed, and new drug treatment was replaced. For the controls, unstained and stained cells were used to set up the compensation, and 1 × 10^6^ cells were grown in a T25 flask. Post-treatment on day 5, cells were collected, washed twice with cold PBS, and stained with Annexin V and PI according to the manufacturer’s instruction. Briefly, cells collected from each well were washed twice with PBS and resuspended in 50 μl master mix containing FITC conjugated Annexin V and Propidium iodide (PI) in binding buffer followed by gentle vertexing and incubation at room temperature (25 °C) for 15 min in the dark. The stained cells were resuspended in 300 μl binding buffer and immediately analysed by the Flow cytometer using standard protocol.

To set up the unstained control and single colour compensations, cells were either unstained or stained with either FITC Annexin or PI. Four different populations of cells were distinguished in this apoptotic assay: Viable cells—that were negative for annexin V and PI; Early apoptotic cells- that were only positive for Annexin V -FITC; Necrotic cells -cells that were only positive for PI, and Dead or late apoptotic cells that were positive for both Annexin V-FITC and PI. The percentages of 4 distinguished cell populations in each quadrant were determined as displayed in the two-colour dot plot analysis for annexin-V and PI.

### Cell count assay

1.5 × 10^4^ cells were seeded in 24-well plates (Costar) and allowed to settle overnight. The cells were treated the following day with the indicated drugs. At the end of treatment, the media was discarded, cells detached from the plate using trypsin, stained with tryphan blue (Thermofisher) and counted using a cell counter (Countess).

### CellTiter-Glo luminescent cell viability assay

To assess cell proliferation, 3000–6000 cells/well depending on the cell line were seeded in a 96-well plate (Costar) and allowed to settle overnight. Cells were treated with the respective drugs combination for 5 days. At the end of the treatment, media was discarded and the Cell Titre-Glo® assay reagent (Promega) was added as described by the manufacturer’s protocol. Plates were incubated in the dark at RT for 10 min before luminescence was measured using Pherastar plate reader or for 30 min if using CLARIOstar plate reader.

### Organoid culture

Breast tumour tissue was processed for organoid formation within 24 h essentially as described in [23]. Briefly, after washing 3 times in ADF^+++^(Advanced DMEM/F12 containing 1x Glutamex, 10 mM Hepes and penicillin/streptomycin, invitrogen) with Primocin. Breast cancer tissue was cut into small pieces and enzymatically digested for 2 h at 37 °C with Collagenase (Sigma) at concentration of 1.5 mg/ml in complete organoid medium. Tissue sections were sheared occasionally and after completion of digestion the suspension was strained over a 100 µM filter (Falcon) and centrifuged at 400 × *g* for 5 min. The resulting pellet was suspended in complete organoid medium containing 10 mg/ml cold basement membrane (Matrigel Matrix, MG, Corning). 50 µl droplets of MG-cell suspension were plated on pre-warmed 24 -well suspension culture plates (Coster, Corning) for 20 min at 37 °C followed by the addition of 500 µl of BC organoid medium and transferred to a humidified 37 °C/5% CO_2_ incubator. The complete breast cancer organoid medium was prepared as follows: ADF^+++^ was supplemented with 250 ng/ml R-Spondin (R &D), 5 nM Neuregulin, 5 ng/ml FGF7, 20 ng/ml FGF10, 5 ng/ml EGF, 100 ng/ml Noggin (all from Peprotech), 500 nM A83-01(Tocris), 5uM Trastuzumab-27632 (Abmole), 500 nM SB202190 (Sigma), 1xB27 supplement (Gibco), 1.25 mM N-Acetylcysteine (Sigma), 5 mM Nicotinamide (Sigma) [23]. Organoid medium was changed every 4 days and organoids were passaged once they developed into structures >100 μm, every 1–2 weeks by incubating in TripLE (Gibco) at 37 °C for 5–10 min to dissociate into single cells and replating in fresh matrigel containing BC medium [23]. For viability assay, 4000 organoids cells/well/100 µl were plated in organoid medium with 5% MG, after splitting organoids into single cell suspension, were plated into clear bottom black opaque 96 well plates (Coaster) previously coated with 15 µl of MG and prewarmed at 37 °C. After 4–5 days when organoids developed into structures were treated with different drug alone or in combination. On the day 3 post-treatment, media was carefully removed, and 2nd treatment was given, prepared in complete organoid medium with 2% MG. The cell viability was assessed after 5 days, using Cell titre-Glo 3D Reagent (Promega). According to the manufacturer’s manual an equal volume of Cell Titre-Glo® Reagent (100 ml) was added per well and plates were shaken for 5 mins prior to the incubation for 30 min at room temperature. Luminescence was recorded using the Pherastar plate reader.

### NK-cell isolation and organoid co-culture

NK cells were extracted from normal PMBC following human NK cell extraction kit (EasySep™, 19055) protocol. Breast cancer organoids were co-cultured with NK cells in 1:5 ratio for 5 days in a clear well white opaque 96 well plates (COSTAR), covered with 15 μl MG, together with stimulatory molecule IL-2 used at 200 IU/ml. At day 5 post-coculture, drugs were added for cell viability studies in the combination and doses as stated. 5 days later luminescence was measured using 3D cell titre Glo (Promega) following the manufacturer’s protocol.

### Supplementary information

Supplementary figure legends

Supplementary figures 1-8

## Data Availability

All the original western blots and raw data are available from the authors upon request.
