# Supplementary material for: Neratinib could be effective as monotherapy or in combination with trastuzumab in HER2-low breast cancer cells and organoid models
Source: Br J Cancer. 2024 Apr 10;130(12):1990–2002. doi: 10.1038/s41416-024-02665-z (PMC11182766; doi:10.1038/s41416-024-02665-z)
Supplement: Supplementary file 1 — Supplementary figure legends [file 41416_2024_2665_MOESM1_ESM.docx]

**Lists of Supplementary Figures**

Supp Figure 1: Assessment of the HER2 status in a panel of breast cancer cell lines

Supp Figure 2: MDA-MB-453 and MDA-MB361 cells are sensitive to low doses of neratinib

Supp Figure 3: MDA-MB-453 and MDA-MB361 cells are sensitive to low doses of neratinib in combination with anti-HER2 treatments

Supp Figure 4: Sensitivity of MDA-MB-453 and MDA-MB361 cells to neratinib in combination with anti- HER2 treatments

Supp Figure 5: Comparing tucatinib with neratinib +/- trastuzumab in MDA-MB-453 and MCF-7 cells as well as HER2-low patient-derived organoid

Supp Figure 6: Sensitivity of neratinib in HER2-low organoid

Supp Figure 7: Histology and receptor status of excision tissue and corresponding patient-derived breast organoids

Supp Figure 8: Co-culture of organoids with NK cells for the combination treatments with either trastuzumab and or neratinib

**Supplementary Figure Legends:**

**Supp Figure 1**: **Assessment of the HER2 status in a panel of breast cancer cell lines.**

(A) Cell pellets from the indicated breast cancer cells were stained with an antibody detecting HER2 protein level using IHC. For FISH, HER2 gene level was assessed using CEP17/HER2 FISH probes. Probes binding to chromosome 17 gave a green signal and probes binding HER2 gave a red signal. (B) Quantification of HER2 by IHC and FISH stained in (A). (C) Cell viability with trastuzumab in panel of breast cancer cells. Cells were seeded in 96-well plates and left to settle overnight before being treated with the indicated doses of trastuzumab for 5 days. Afterwards medium was discarded and the cell titre blue substrate added for 2h before analysis.

**Supp Figure 2**: **MDA-MB-453 and MDA-MB361 cells** **are sensitive to low doses of neratinib and neratinib induces HER2 downregulation via dissociation of HSP90 from HER2.** (A) MDA-MB361 and (B) MDA-MB-453 cells were treated with low dose of neratinib, 5nM, 40 µg/ml trastuzumab and 20 µg/ml of pertuzumab for 24 hours either alone or in combination. Cell lysates were collected and used for western blot as indicated. (C) MDA-MB-453 cell was treated with 1 µg/ml DMSO and 10nM neratinib 10nM for 24 hours. HSP90 was immunoprecipitated from cell lysates, then the samples were analysed by immunoblotting with anti-HER2 and anti-HSP90 antibodies.

**Supp Figure 3**: MDA-MB-453 and MDA-MB361 cells **are sensitive to low doses of neratinib in combination with anit-HER2 treatments**. (A) MDA-MB-361 and (B) MDA-MB-453 cells were treated with low dose 5nM neratinib, 40 µg/ml trastuzumab and 20 µg/ml of pertuzumab for 24 hours alone or in combination as indicated. Cell lysates were used for western blot as indicated. B-actin used as a loading control for 3 independents experiment, representative actin blot is shown.

**Supp Figure 4: Sensitivity of** MDA-MB-453 and MDA-MB361 cells **to neratinib in combination with anti-HER2 treatments.** (A) MDA-MD-361 and MDA-MB-453 (B) cells were treated with 10 nM neratinib, 40 ug/ml trastuzumab and 20 µg/ml of pertuzumab for 24 hours in serum-reduced media and viable cells were assessed using an automated cell counter using the trypan blue exclusion test. DMSO + IgG were used as a control. Graphs plotted represent the mean ± SEM. Difference in the mean between groups was analysed by one-way Anova with Turkey multiple comparison test; statistically significant changes are represented by asterisks as *p ≤ 0.05, **p ≤ 0.01, ***p ≤ 0.001 and ****p < 0.0001. (C) MDA-MB-361 cells were treated for 5 days in 5% FBS and stained for Annexin V and the percentage of early apoptotic cells and dead cells was assessed by FACS analysis. Two-way ANOVA - Turkey multiple comparison test was used to determine the significance between the two treatment groups. P values are denoted as *p ≤ 0.05, **p ≤ 0.01, ***p ≤ 0.001 and ****p < 0.0001. All data is presented as the mean ± SEM from 3 independent experiments (n=3) with 3 technical repeats. (D) MDA-MB-361 and MDA-MB-453 cells were treated with increasing concentrations of neratinib and neratinib in combination with 40 ug/ml of trastuzumab for 5 days. Viable cells were assed using 2D cell titre Glo assay and the graph plotted is normalized to DMSO (control). Two-way ANOVA - Turkey multiple comparison test was used to determine the significance between the two treatment groups. P values are denoted as *p ≤ 0.05, **p ≤ 0.01, ***p ≤ 0.001 and ****p < 0.0001. All data is presented as the mean ± SEM from 3 independent experiments (n=3) with 3-4 technical repeats.

**Supp Figure 5: Comparing tucatinib with neratinib +/- trastuzumab in MDA-MB-453 and MCF-7 cells as well as HER2-low patient-derived organoid.** (A) MDA-MB-453 cells were treated with 1 µg/mL IgG , 1 µg/ml DMSO, 10nM neratinib or 10nM tucatinib 4 hours. The cell lysates were used for western blot to analyse the proteins as indicated. (B) MCF-7 and MDA-MB-453 cells were treated with 1 µg/ml IgG , 1 µg/mL DMSO, 40 µg/ml trastuzumab, 10nM neratinib or 10nM tucatinib as monotherapy or in combination for 24 hours. The cell lysates were used to analyse the indicated proteins by western blot. (C) MCF-7 and MDA-MB-453 cells were seeded in a 96- well plate at a density of 8000 cells per well and left to adhere for 24 hours. Cells were then treated with 1 µg/ml IgG , 1 µg/ml DMSO, 40 µg/ml trastuzumab, 10nM neratinib or 10nM of tucatinib as monotherapy or in combination for 5 days. The cell viabilities were obtained by CellTiter-Glo® Luminescent Cell Viability Assay and the data were normalized to control (IgG + DMSO). Data are presented as the means +/- the standard deviation from 3 independent experiments with 3 technical repeats. The difference in the mean of each group was assessed by the one-way ANOVA with Turkey’s multiple comparison test; statistically significant differences are represented by asterixis as *p ≤ 0.05, ** p ≤ 0.01, ***p ≤ 0.001 and ****p < 0.0001. (D) T-485871 patient-derived HER2-low breast cancer organoids were seeded at a density of 6,000 cells per organoid dome, and were left to grow into structures for 5 days. Organoids were treated with either DMSO as a control, 100nM Neratinib, or 100nM Tucatinib, and incubated for a further 5 days. Cell viability was assessed using a 3D viability assay. Samples are normalised to the DMSO control, to account for cell number due to DMSO toxicity, with data presented as the mean + SD. Statistical analysis was performed using a one-way ANOVA, where ***p ≤ 0.001 and ****p < 0.0001. All data is presented as the mean ± SEM from 3 independent experiments (n=3) with multiple technical repeats.

**Supp Figure 6: Histology and receptor status of excision tissue and corresponding patient-derived breast organoids.**

(A) Breast cancer organoids architecture resembles their histology epithelium. H&E staining of tumour biopsy and its derived organoids have resembling tubular structures and organoids show the presence of two lumens recapitulating tumour features. The core biopsy of normal tissue (on right) displays tubules, and its corresponding organoid resembles mitotic cluster and absence of lumen. Scale bars = 100 μM

(B) Comparative histological and immunohistochemical images of tumour tissue (left) and corresponding patient derived organoids (right). Shown are the representative example of no special type HER2 positive breast carcinoma. Tissue (left) presents with characteristics of tumour epithelium and surrounded by inflammatory cells, while organoids (right) recapitulate tumour epithelium (H&E). The excision tissue derived organoids recapitulate the expression of immunohistochemical markers; ER, PR, and HER2 status. The occasional ER and PR negative cells found in the HER2 positive excision tissue are retained in the derived organoids. Scale bars = 100 μM

(C) Comparative histological images of normal mammary lobule (left) and its respectively derived organoid (right). Representative example from the normal core biopsy tissue resembles the corresponding organoids. The immunohistochemical status of ER, PR, and HER2 is generally maintained in derived organoids. Scale bars = 100 μM

(D) Bright-field images depicting breast cancer organoid phenotypes. Corresponding organoid TS403276 shows dense and solid structures, while NS403275 shows loose and hollow structures and TS396358 shows thin-walled discohesive organoids.

**Supp Figure 7**: **Sensitivity of neratinib in HER2-low breast cancer organoids.** (A) T-S396358 and (B) T-S358174 organoids were grown in 96 well plate with 2.5% Matrigel. T-S396358 PDOs were treated with an increasing concentration of neratinib alone and in combination with 40 ug/ml trastuzumab for 5 days as indicated. (C) T-S358174 PDOs were treated with a higher concentration of neratinib as a monotherapy and or in combination with 40 ug/ml trastuzumab as indicated. Cell proliferation was assessed using 3D cell titre Glo and the graph was normalized to the control (DMSO + IgG). Representative pictures (x 20) for each treatment on day 1 and day 5 are shown and the scale bars represent 100 μm. Data is presented as the median from 3 independent experiments with 3-4 technical replicates. Non-parametric Kruskal Wallis test was performed; p values are denoted as *p ≤ 0.05, ** p ≤ 0.01, ***p ≤ 0.001 and ****p < 0.0001.

**Supp Fig 8: Co-culture of organoids with NK cells for the combination treatments with either trastuzumab and or neratinib.** PDOs T-S403276 (HER2+ FISH positive ER/PR+) and T-S446358 (HER2 0 ER/PR+) were co-cultured with NK cells in 2.5% Matrigel in the 96 well plate. NK cells were stimulated for 24 hours with IL-2 to allow activation of NK cells prior to treatment with the drugs. Organoids and NK cells co-cultures were treated with different doses of (A) Neratinib and (B) trastuzumab for 5 days before luminescence was measured using 3D cell titre Glo and the results were normalized to the control (DMSO +IgG). Data presented from three independent experiments each with 3-4 technical replicates; data is shown as median ± range; non-parametric Kruskal Wallis test was performed and p values are denoted as *p ≤ 0.05, ** p ≤ 0.01, ***p ≤ 0.001 and ****p < 0.0001. (C) PDOs were treated with neratinib and trastuzumab alone or in combination for 5 days with ± NK cells. Viable cells were assed using 3D cell titre Glo assay and the graph plotted is normalized to DMSO (control) Two-way ANOVA - Turkey multiple comparison test was used to determine the significance between the two treatment groups. P values are denoted as *p ≤ 0.05, ** p ≤ 0.01, ***p ≤ 0.001 and ****p < 0.0001. All data is presented as the median ± range from 3 independent experiments with 3-4 technical repeats. (D) NK-cells were treated with anti-CD16 and co-cultured with PDO T- S403276 followed by treatment with neratinib and trastuzumab alone or in combination for 5 days with ± NK cells. All data is presented as the median ± range from 3 independent experiments with 3-4 technical repeats. Two-way ANOVA - Turkey multiple comparison test was used, and P values are denoted as *p ≤ 0.05, ** p ≤ 0.01, ***p ≤ 0.001 and ****p < 0.0001.
