# Supplementary material for: Neratinib could be effective as monotherapy or in combination with trastuzumab in HER2-low breast cancer cells and organoid models
Source: Br J Cancer. 2024 Apr 10;130(12):1990–2002. doi: 10.1038/s41416-024-02665-z (PMC11182766; doi:10.1038/s41416-024-02665-z)
Supplement: Supplementary file 2 — Supplementary figures 1-8 [file 41416_2024_2665_MOESM2_ESM.pptx]

## Slide 1
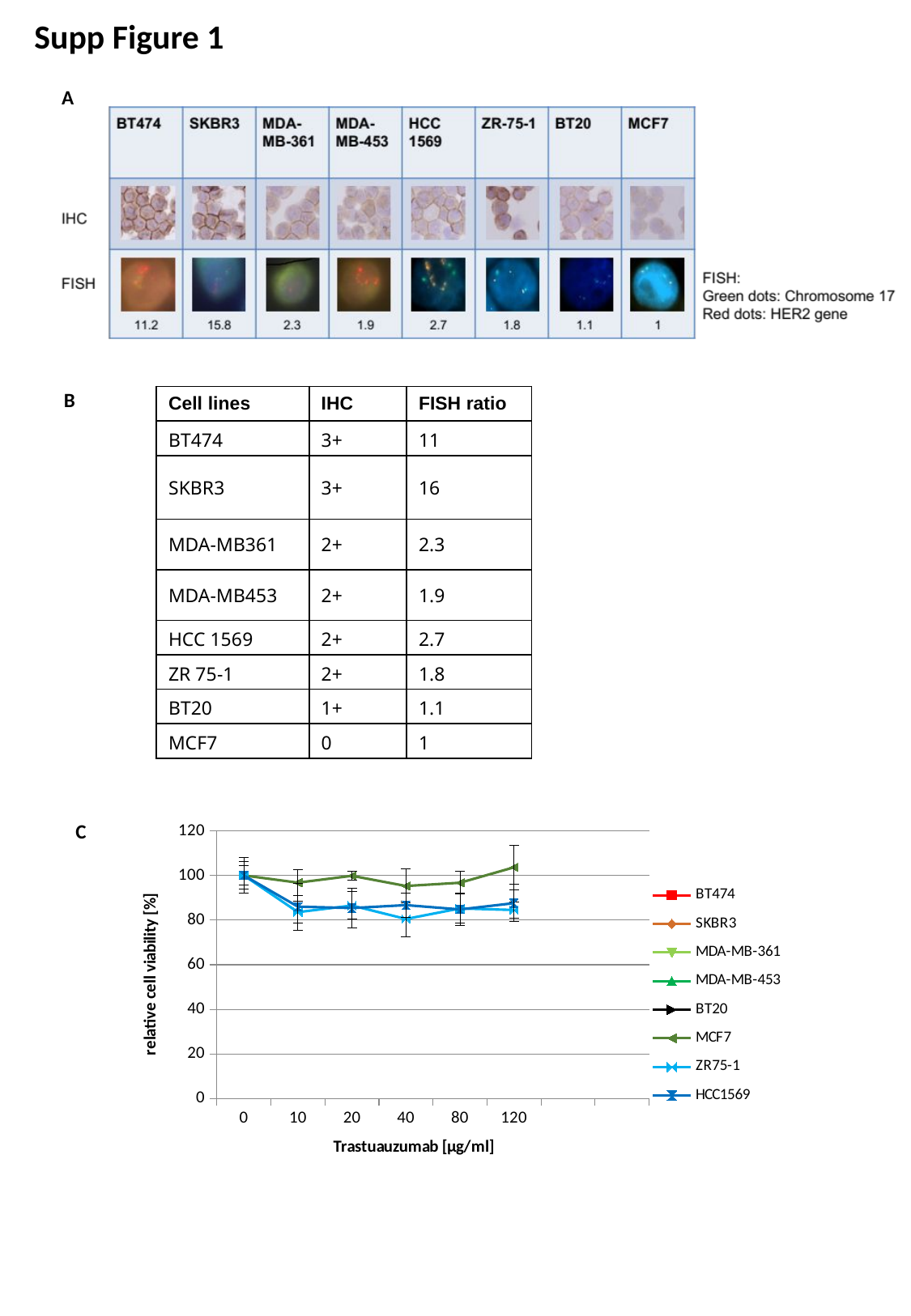

Supp Figure 1
A
B
| Cell lines | IHC | FISH ratio |
| --- | --- | --- |
| BT474 | 3+ | 11 |
| SKBR3 | 3+ | 16 |
| MDA-MB361 | 2+ | 2.3 |
| MDA-MB453 | 2+ | 1.9 |
| HCC 1569 | 2+ | 2.7 |
| ZR 75-1 | 2+ | 1.8 |
| BT20 | 1+ | 1.1 |
| MCF7 | 0 | 1 |
C
### Chart
| Category | BT474 | SKBR3 | MDA-MB-361 | MDA-MB-453 | BT20 | MCF7 | ZR75-1 | HCC1569 |
|---|---|---|---|---|---|---|---|---|
| 0 | 100.0 | 100.0 | 100.0 | 100.0 | 100.0 | 100.0 | 100.0 | 100.0 |
| 10 | 38.76019247519015 | 49.16910812767173 | 67.8085453458565 | 58.987398994179806 | 104.47011721220144 | 96.75373808142854 | 83.56012219051713 | 86.01747133939718 |
| 20 | 37.07850153139653 | 44.99860887800046 | 70.47446408521212 | 54.31501564436951 | 101.58116924179018 | 99.81154988864894 | 86.51184112172938 | 85.4016849360225 |
| 40 | 33.60170097994312 | 46.49054844650556 | 63.19313380622234 | 58.53752023366792 | 104.58006423269256 | 95.26045036595036 | 80.48193541379085 | 86.70365263386525 |
| 80 | 37.19086036013457 | 44.80139152847885 | 70.53359823960753 | 62.473265555233866 | 97.33076144081367 | 96.70994667638986 | 85.21050905441653 | 84.76242230937054 |
| 120 | 38.666679145545906 | 46.64857263181134 | 65.23980189137184 | 60.13428604718265 | 106.7361243813749 | 103.63163271218758 | 84.5162323082105 | 87.56710474813092 |

## Slide 2
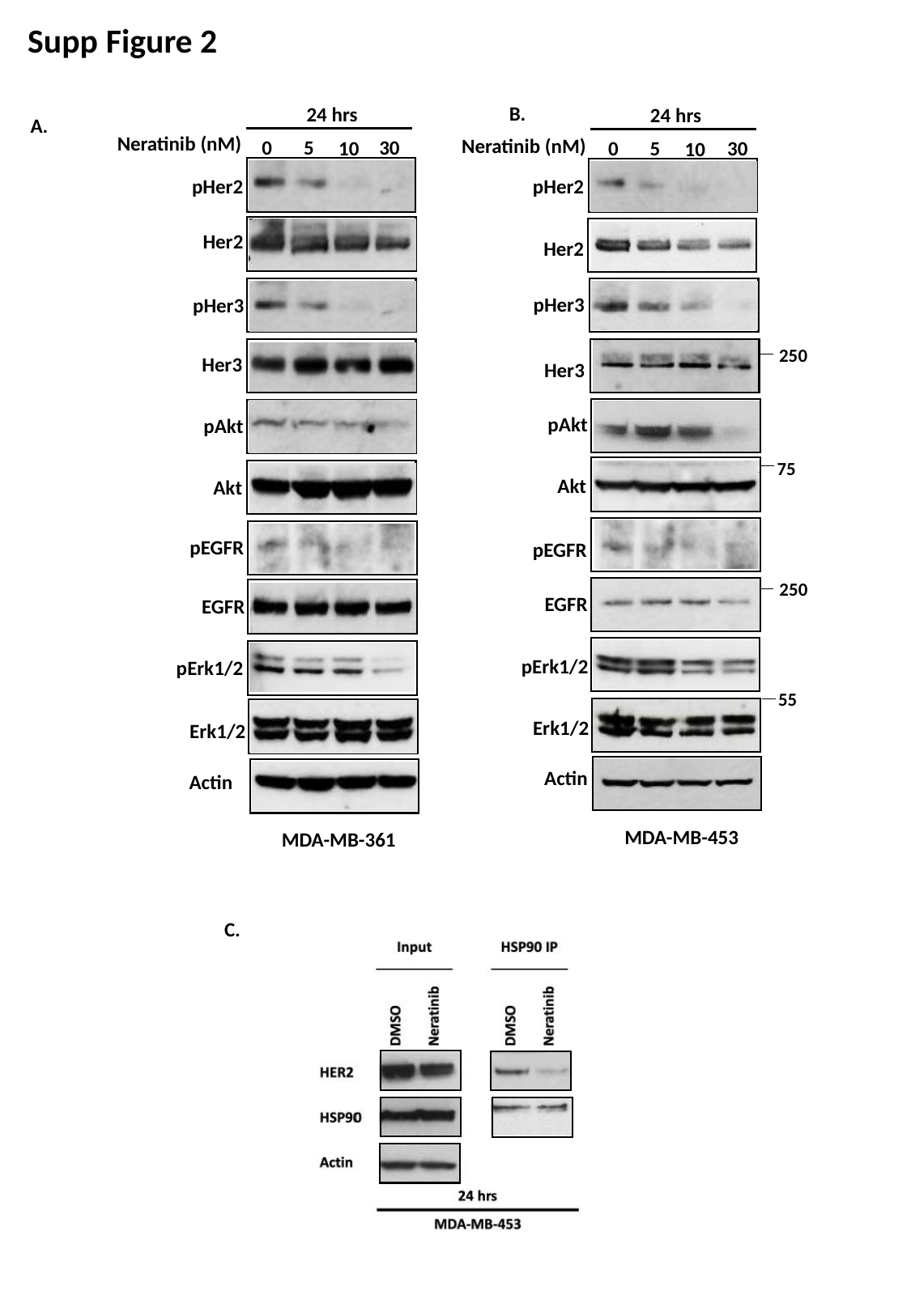

Supp Figure 2
B.
24 hrs
A.
Neratinib (nM)
5
30
0
10
pHer2
Her2
pHer3
Her3
pAkt
Akt
pEGFR
EGFR
Erk1/2
Actin
MDA-MB-361
24 hrs
Neratinib (nM)
5
30
0
10
pHer2
Her2
pHer3
250
Her3
pAkt
75
Akt
pEGFR
250
EGFR
pErk1/2
55
Erk1/2
Actin
MDA-MB-453
pErk1/2
C.

## Slide 3
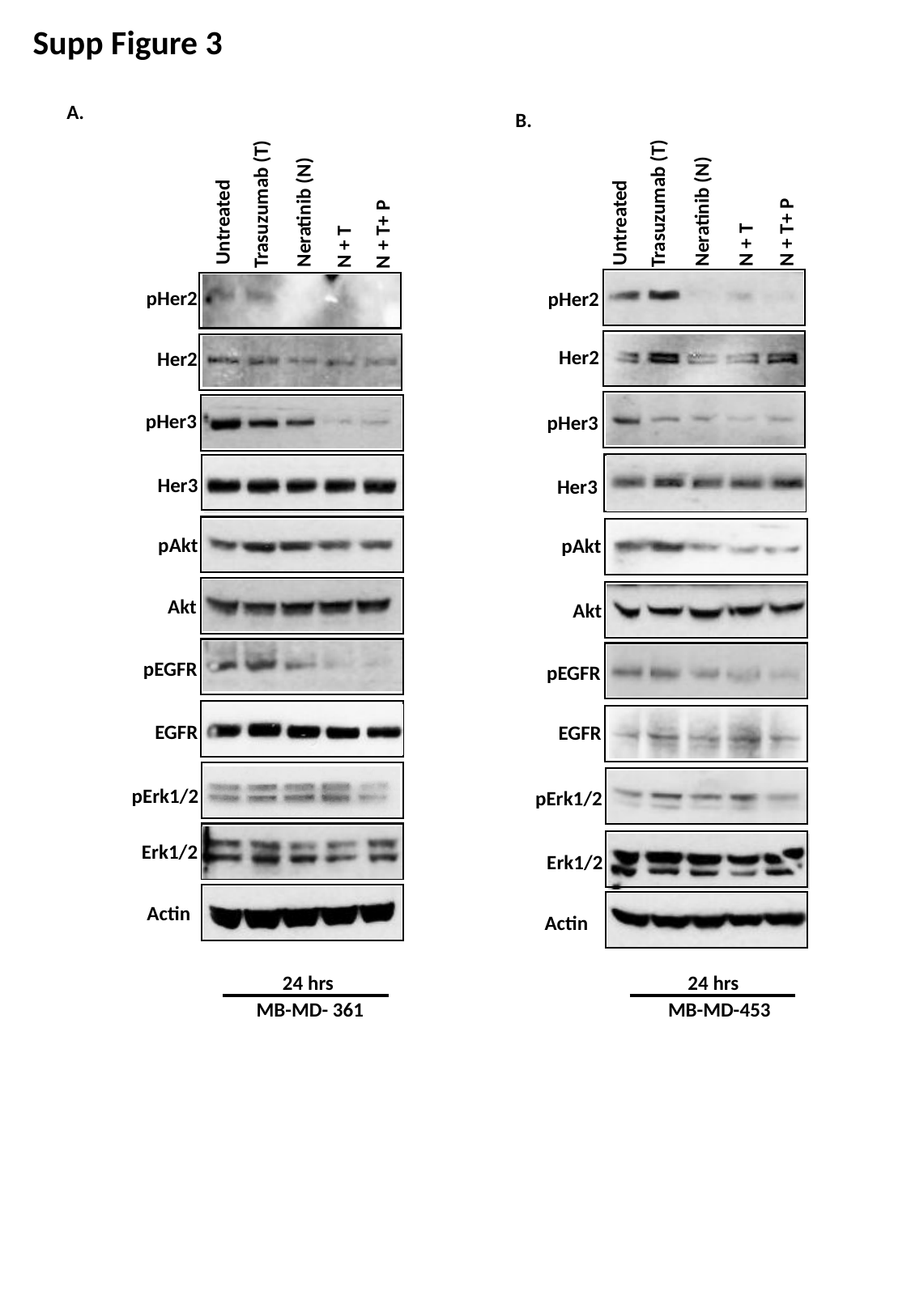

Supp Figure 3
A.
B.
Neratinib (N)
Trasuzumab (T)
Untreated
N + T+ P
N + T
pHer2
Her2
pHer3
Her3
Her3
pAkt
Akt
pEGFR
EGFR
pErk1/2
Erk1/2
24 hrs
MB-MD-453
Neratinib (N)
Trasuzumab (T)
N + T+ P
N + T
Untreated
pHer2
Her2
pHer3
Her3
pAkt
Akt
pEGFR
EGFR
pErk1/2
Erk1/2
Actin
24 hrs
MB-MD- 361
Actin

## Slide 4
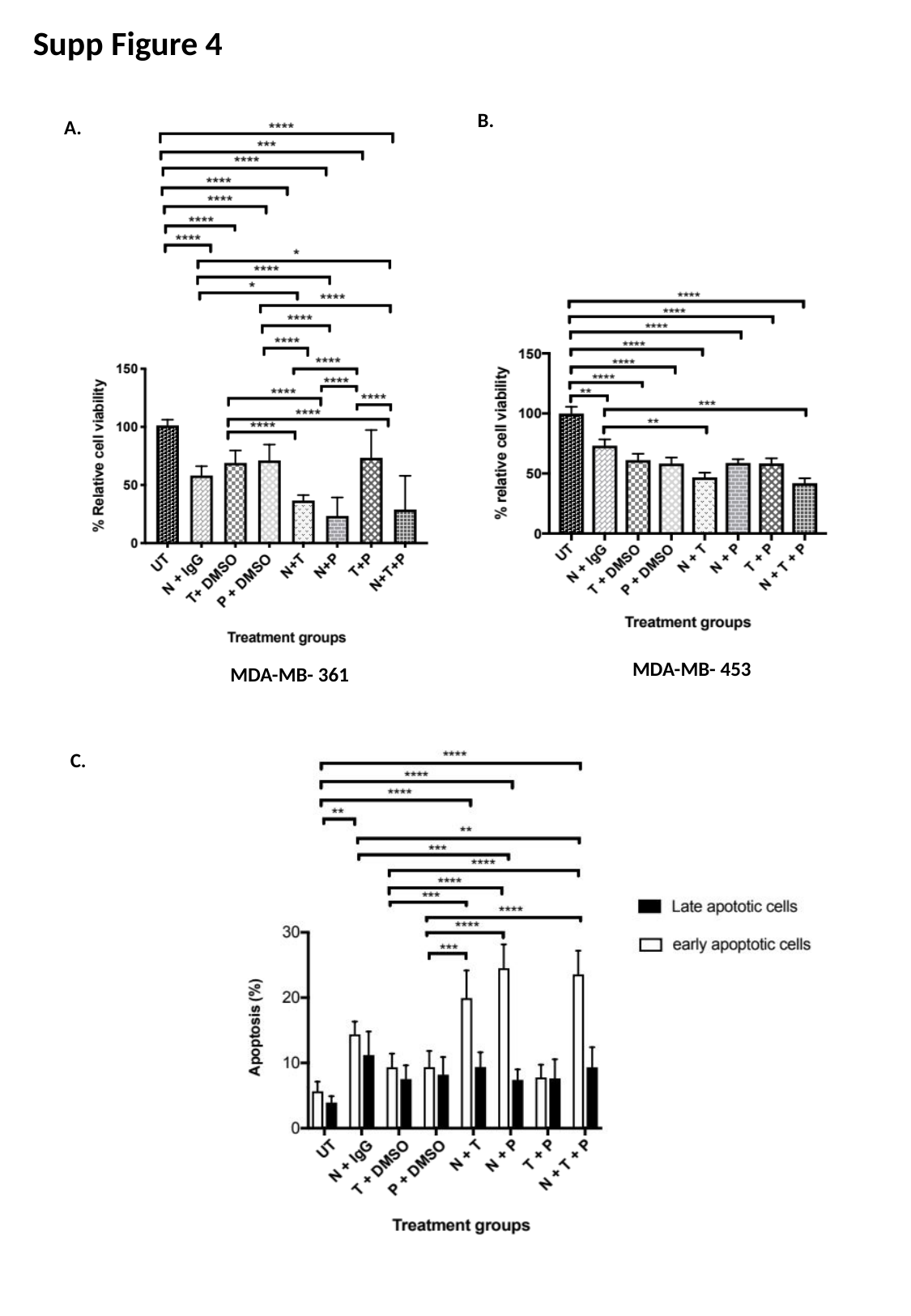

Supp Figure 4
B.
A.
MDA-MB- 453
MDA-MB- 361
C.

## Slide 5
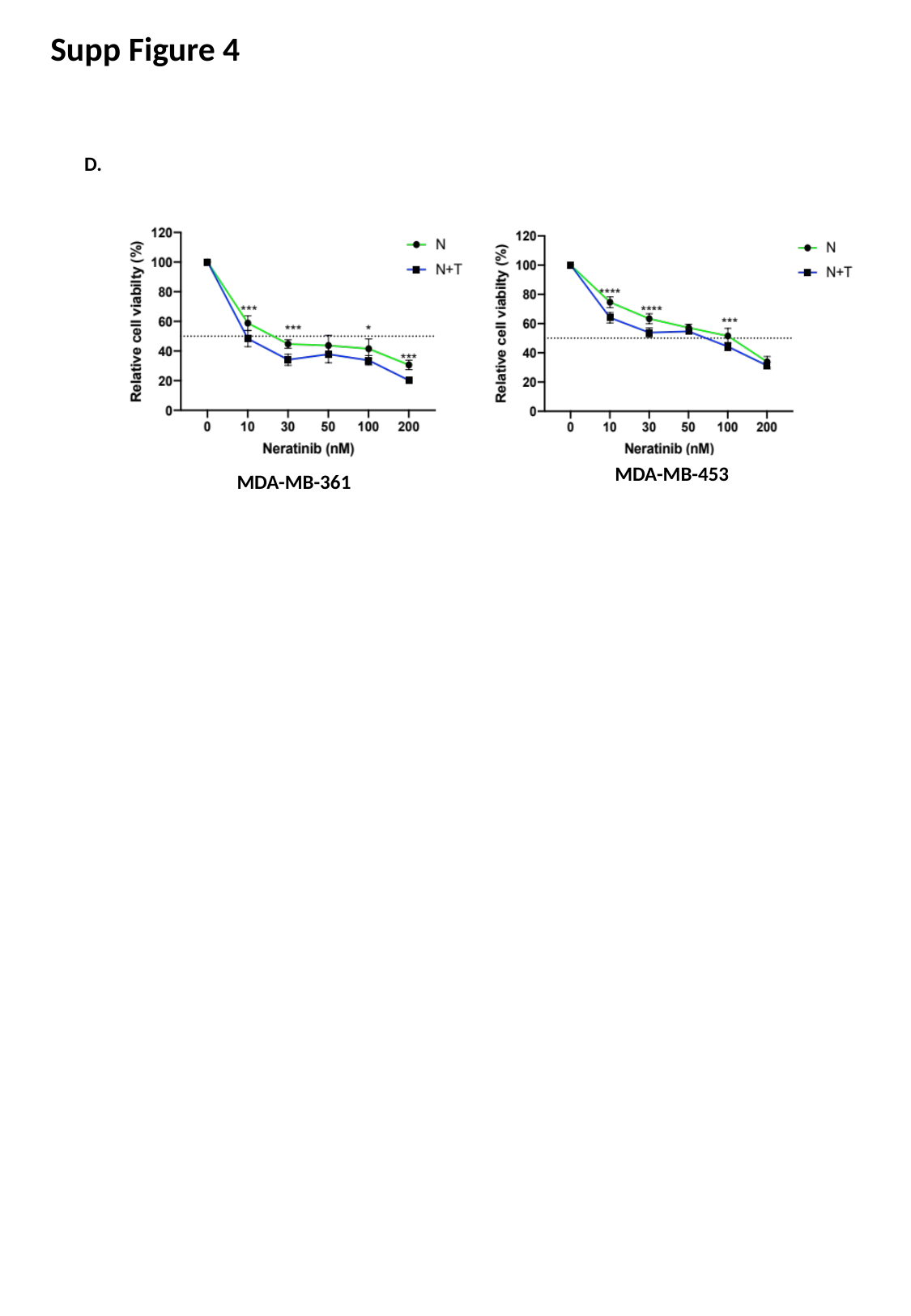

Supp Figure 4
D.
MDA-MB-361
MDA-MB-453

## Slide 6
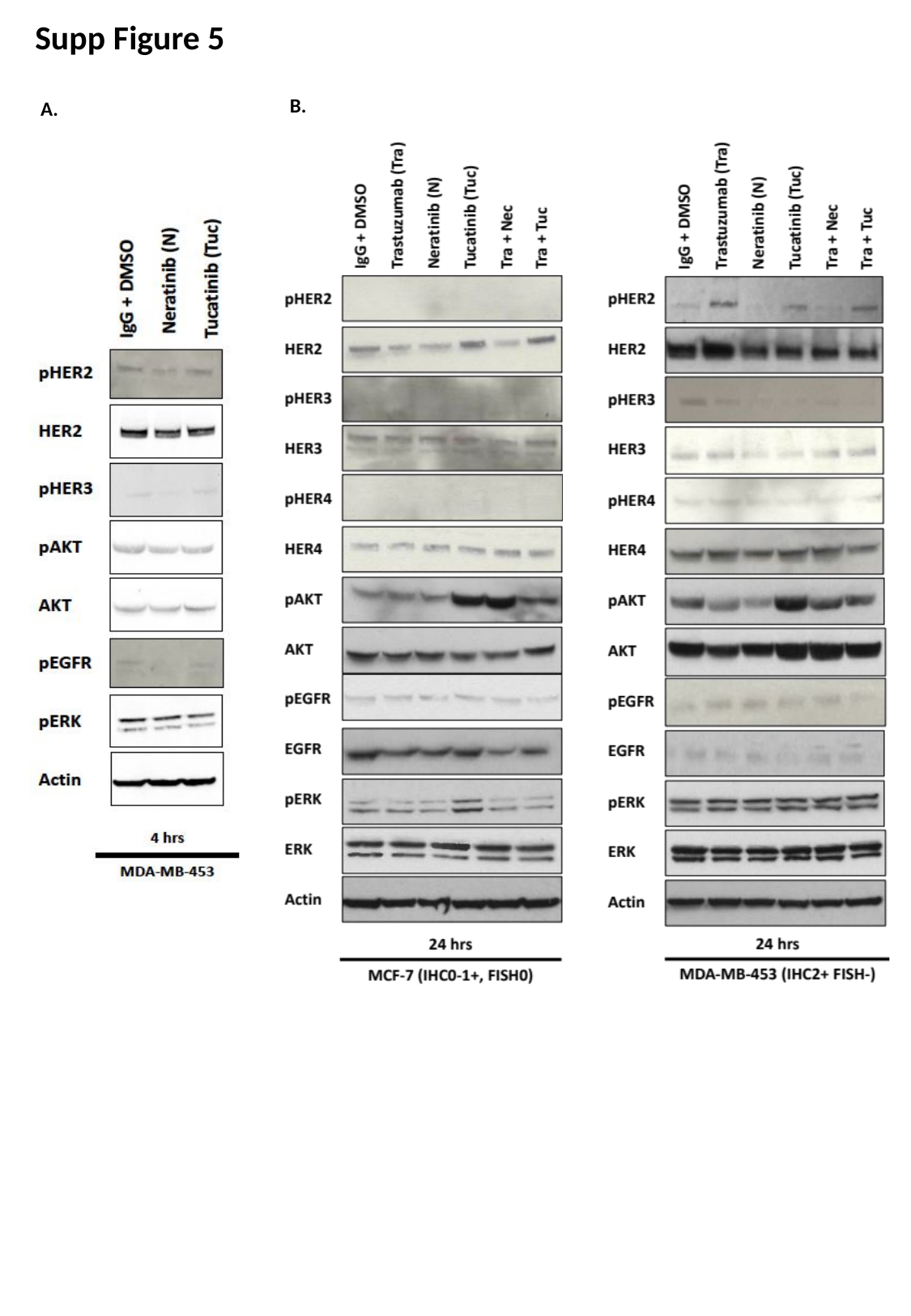

Supp Figure 5
B.
A.

## Slide 7
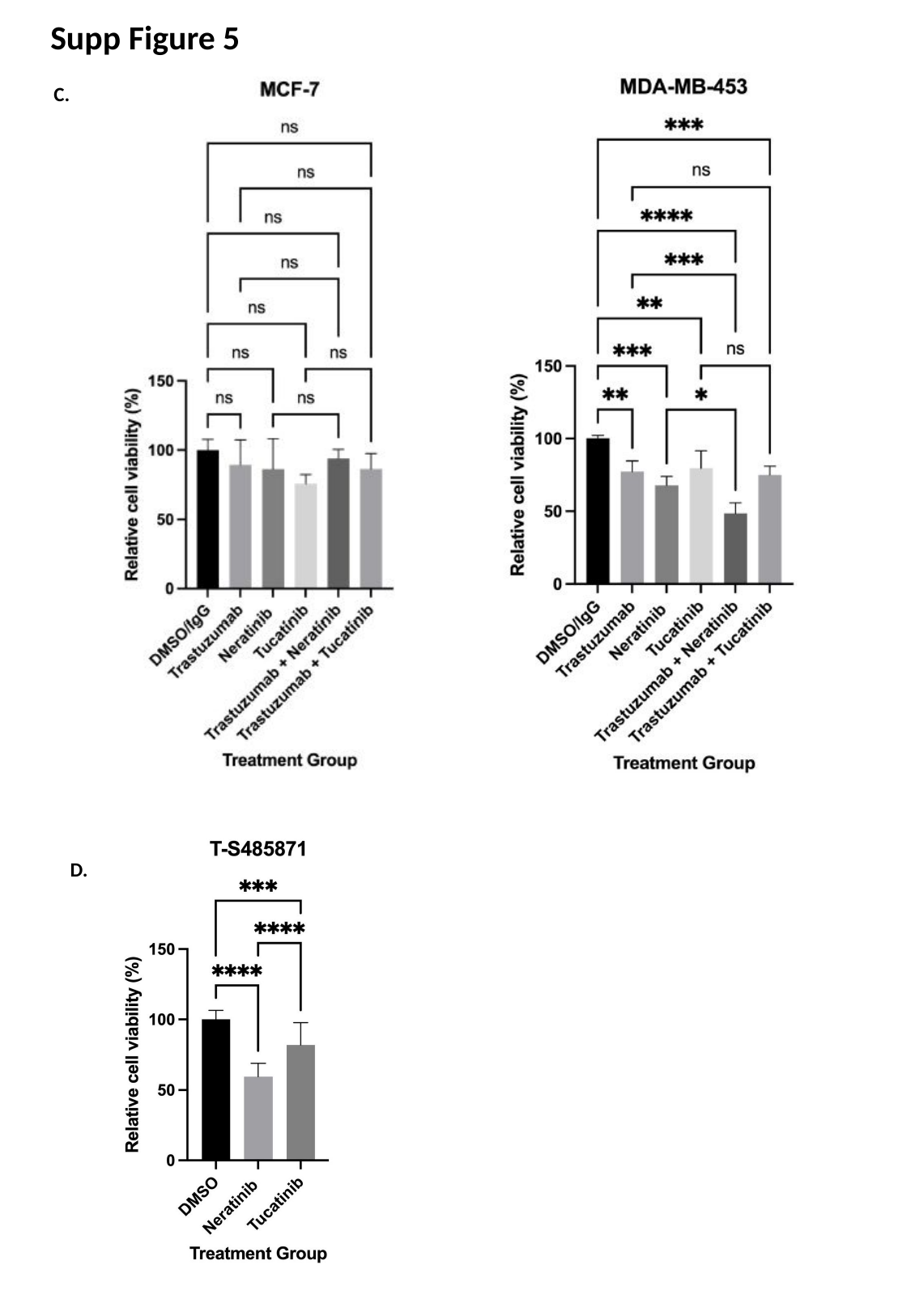

Supp Figure 5
C.
D.

## Slide 8
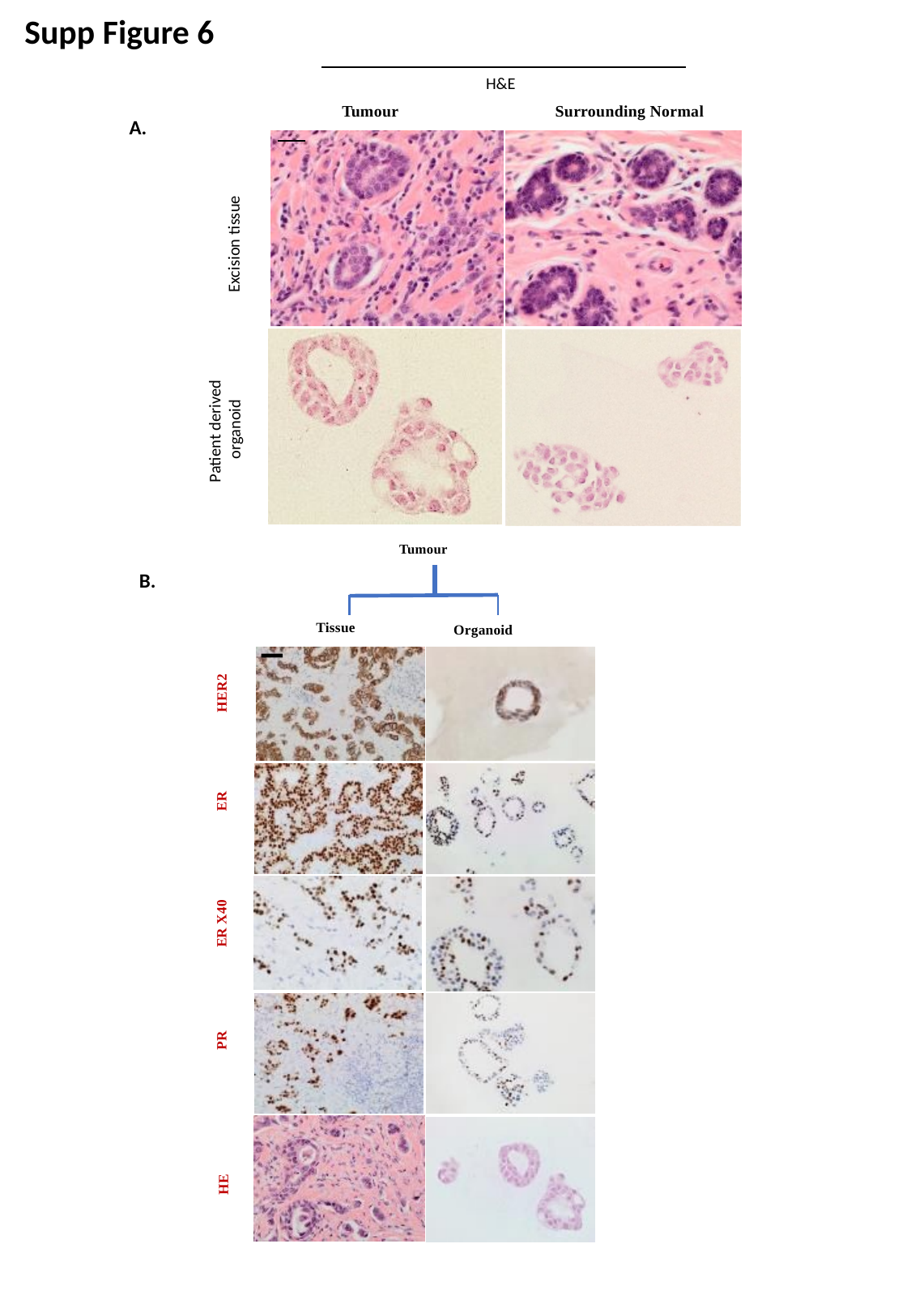

Supp Figure 6
H&E
Surrounding Normal
Tumour
Excision tissue
Patient derived
 organoid
A.
Tumour
Tissue
Organoid
HER2
ER
ER X40
PR
HE
B.

## Slide 9
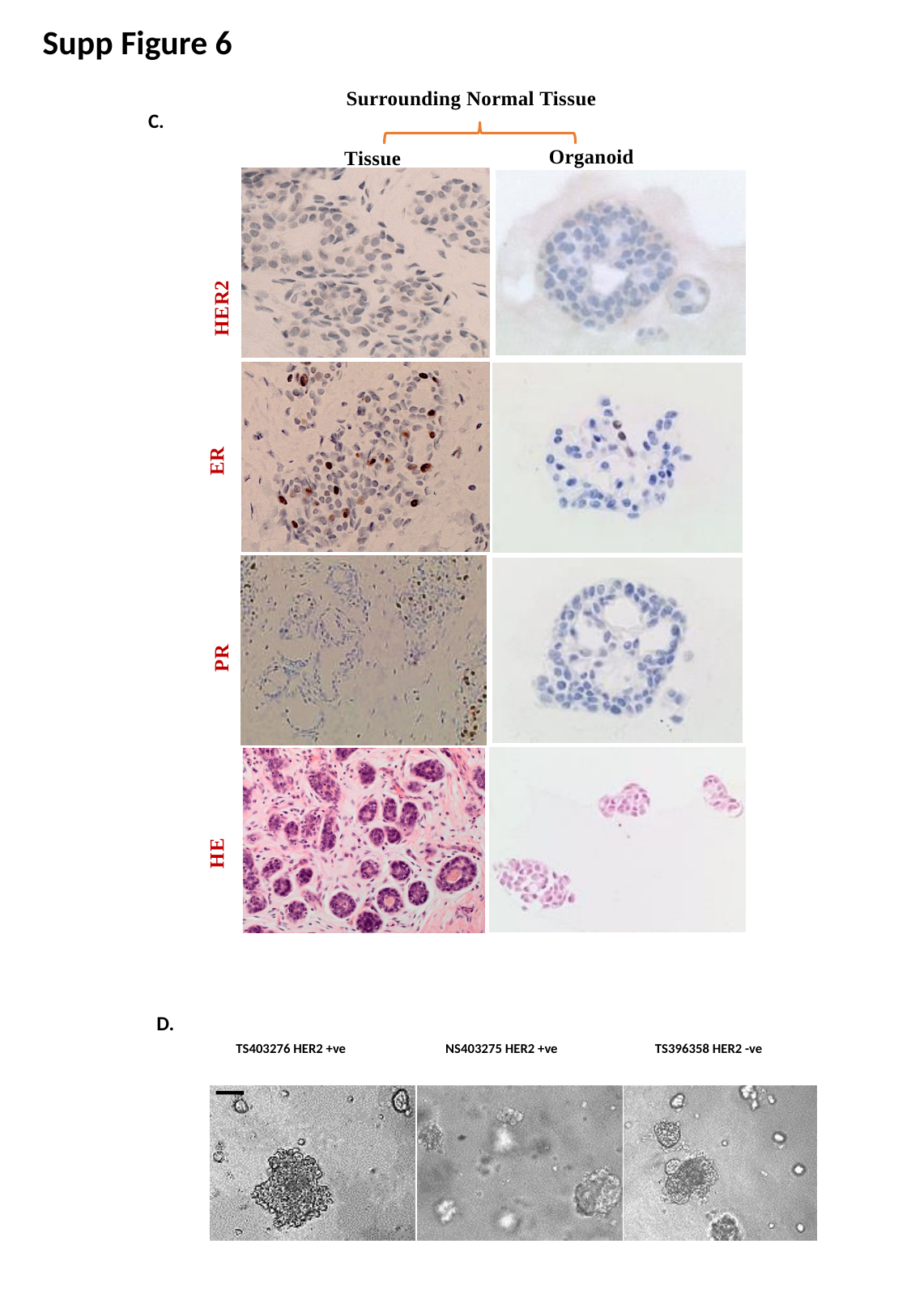

Supp Figure 6
Surrounding Normal Tissue
Organoid
Tissue
HER2
ER
PR
HE
C.
D.
TS403276 HER2 +ve
NS403275 HER2 +ve
TS396358 HER2 -ve

## Slide 10
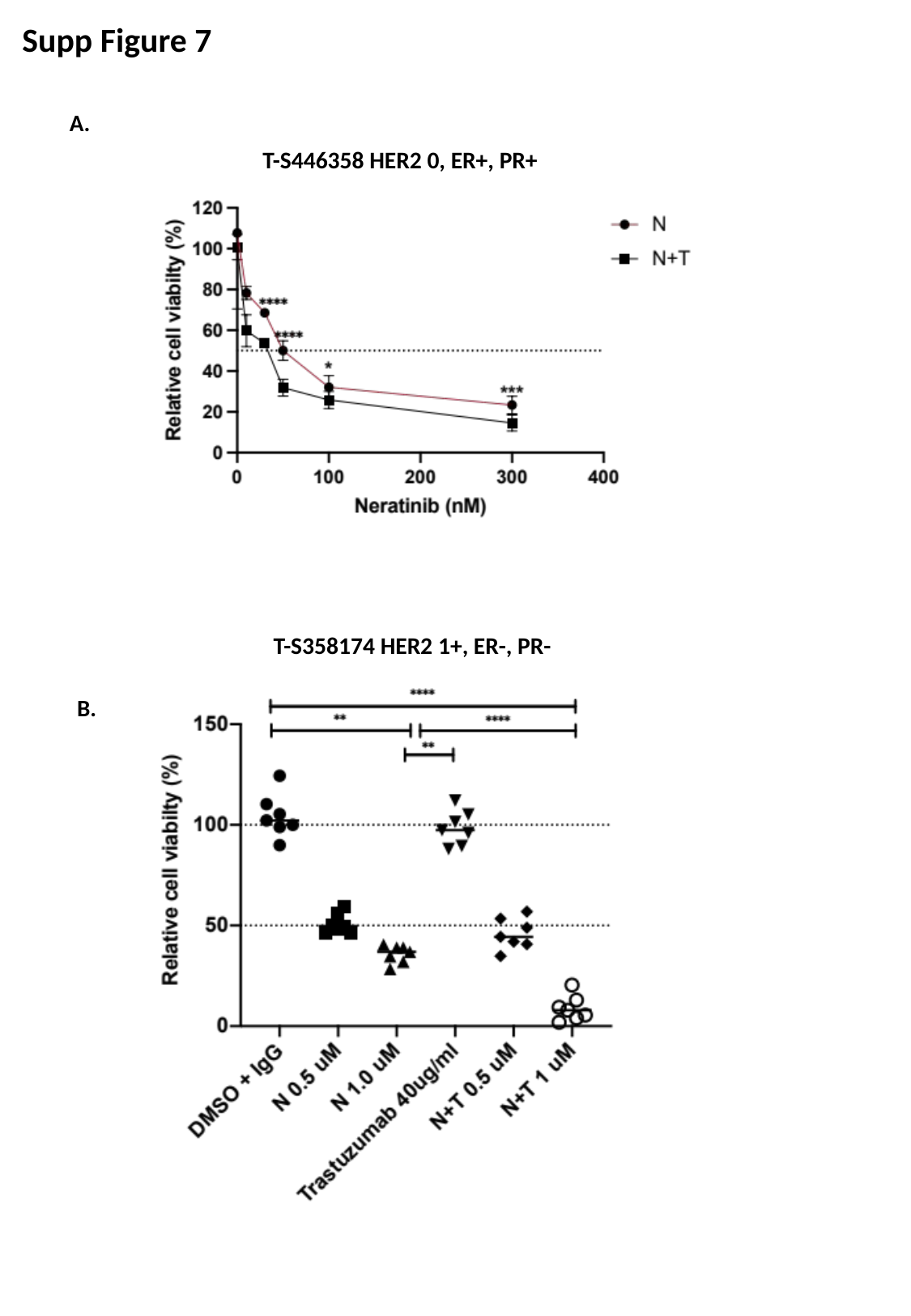

Supp Figure 7
A.
T-S446358 HER2 0, ER+, PR+
T-S358174 HER2 1+, ER-, PR-
B.

## Slide 11
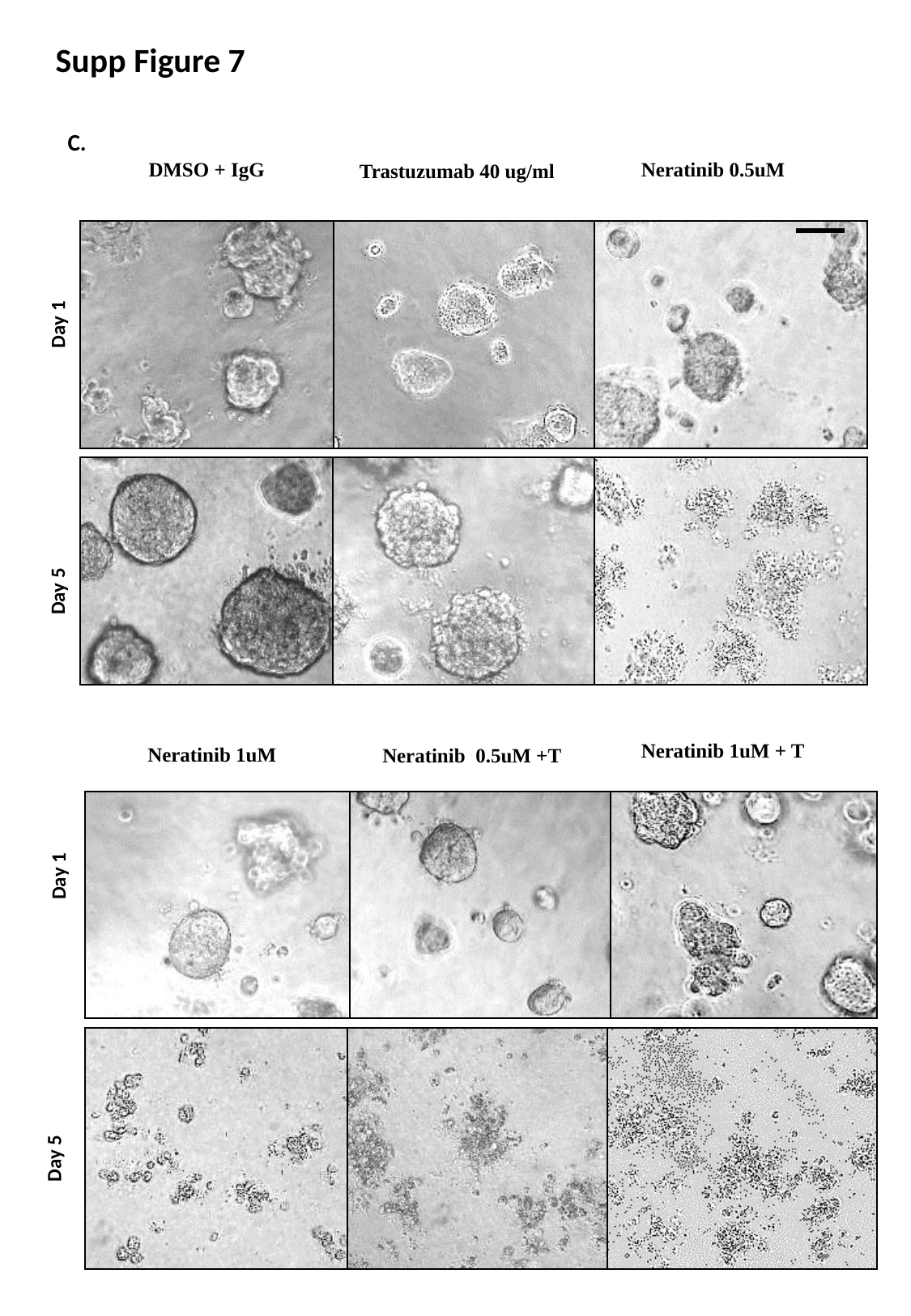

Supp Figure 7
C.
DMSO + IgG
Neratinib 0.5uM
Trastuzumab 40 ug/ml
Day 1
Day 5
Neratinib 1uM + T
Neratinib 1uM
Neratinib 0.5uM +T
Day 1
Day 5

## Slide 12
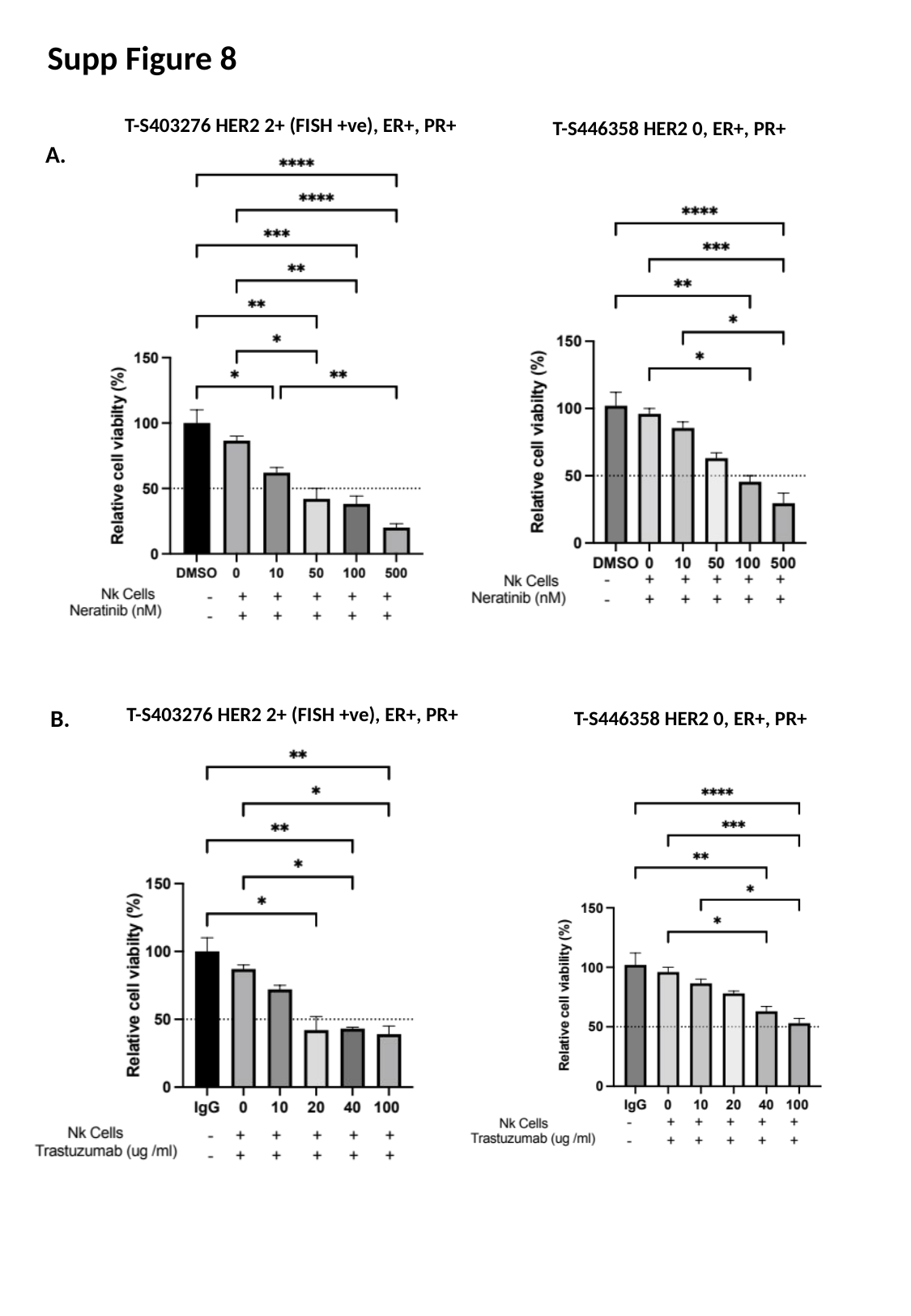

Supp Figure 8
T-S403276 HER2 2+ (FISH +ve), ER+, PR+
T-S446358 HER2 0, ER+, PR+
A.
T-S403276 HER2 2+ (FISH +ve), ER+, PR+
B.
T-S446358 HER2 0, ER+, PR+

## Slide 13
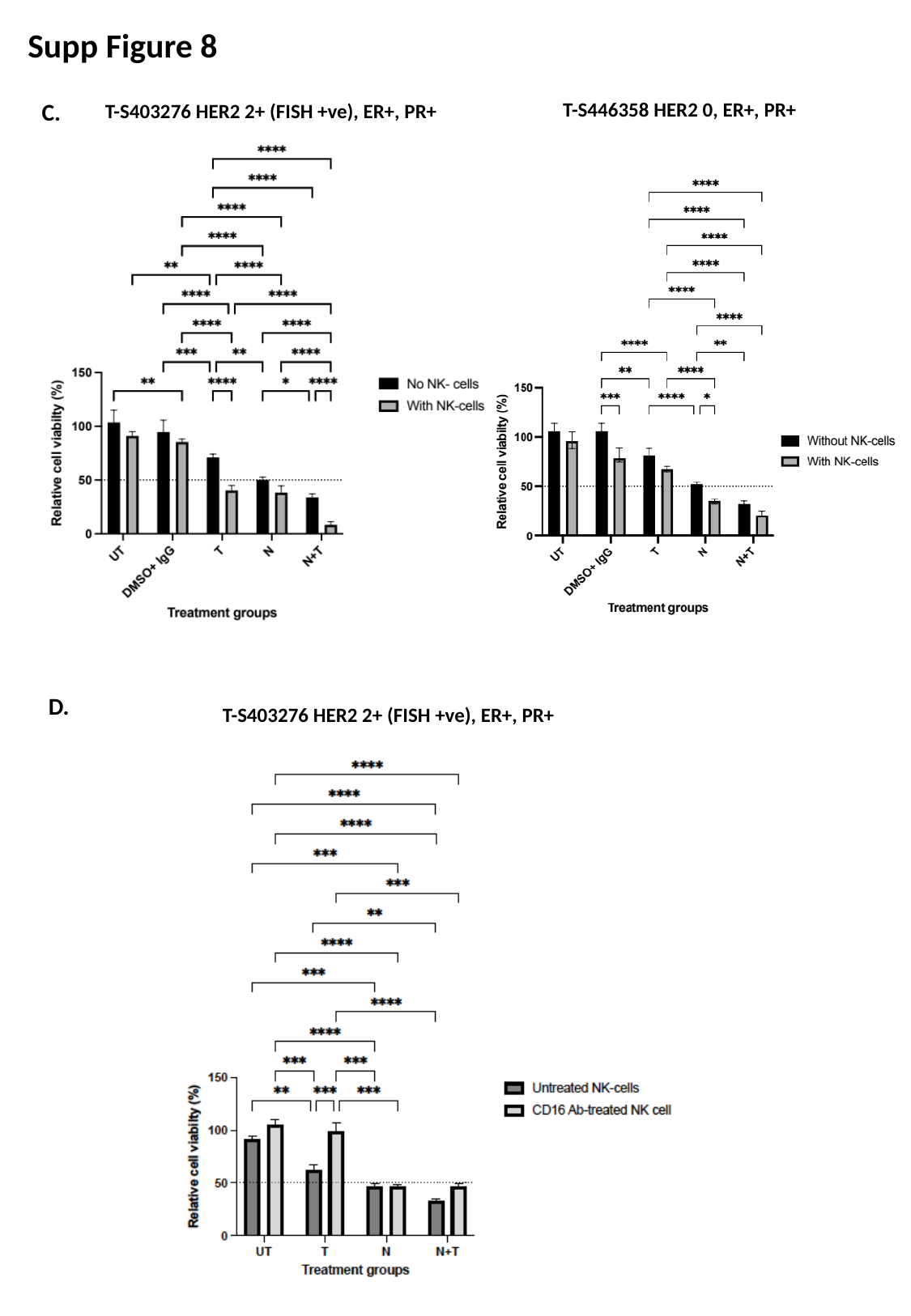

Supp Figure 8
T-S446358 HER2 0, ER+, PR+
C.
T-S403276 HER2 2+ (FISH +ve), ER+, PR+
D.
T-S403276 HER2 2+ (FISH +ve), ER+, PR+
